# Cross-reactive probes on Illumina DNA methylation arrays: a large study on ALS shows that a cautionary approach is warranted in interpreting epigenome-wide association studies

**DOI:** 10.1093/nargab/lqaa105

**Published:** 2020-12-17

**Authors:** Paul J Hop, Ramona A J Zwamborn, Eilis J Hannon, Annelot M Dekker, Kristel R van Eijk, Emma M Walker, Alfredo Iacoangeli, Ashley R Jones, Aleksey Shatunov, Ahmad Al Khleifat, Sarah Opie-Martin, Christopher E Shaw, Karen E Morrison, Pamela J Shaw, Russell L McLaughlin, Orla Hardiman, Ammar Al-Chalabi, Leonard H Van Den Berg, Jonathan Mill, Jan H Veldink

**Affiliations:** Department of Neurology, UMC Utrecht Brain Center, 3584 CG, Utrecht, the Netherlands; Department of Neurology, UMC Utrecht Brain Center, 3584 CG, Utrecht, the Netherlands; University of Exeter Medical School, University of Exeter, Exeter EX2 5DW, UK; Department of Neurology, UMC Utrecht Brain Center, 3584 CG, Utrecht, the Netherlands; Department of Neurology, UMC Utrecht Brain Center, 3584 CG, Utrecht, the Netherlands; University of Exeter Medical School, University of Exeter, Exeter EX2 5DW, UK; Department of Basic and Clinical Neuroscience, King’s College London, Maurice Wohl Clinical Neuroscience Institute, London SE5 9RS, UK; Department of Biostatistics and Health Informatics, King’s College London, London SE5 8AF, UK; Department of Basic and Clinical Neuroscience, King’s College London, Maurice Wohl Clinical Neuroscience Institute, London SE5 9RS, UK; Department of Basic and Clinical Neuroscience, King’s College London, Maurice Wohl Clinical Neuroscience Institute, London SE5 9RS, UK; Department of Basic and Clinical Neuroscience, King’s College London, Maurice Wohl Clinical Neuroscience Institute, London SE5 9RS, UK; Department of Basic and Clinical Neuroscience, King’s College London, Maurice Wohl Clinical Neuroscience Institute, London SE5 9RS, UK; Department of Basic and Clinical Neuroscience, King’s College London, Maurice Wohl Clinical Neuroscience Institute, London SE5 9RS, UK; UK Dementia Research Institute, King’s College London, London WC2R 2LS, UK; Faculty of Medicine, Health & Life Sciences, Queen’s University Belfast, 90 Lisburn Road, Belfast, BT9 6AG, Northern Ireland, UK; Sheffield Institute for Translational Neuroscience, University of Sheffield, Sheffield S10 2HQ, UK; Complex Trait Genomics Laboratory, Smurfit Institute of Genetics, Trinity College Dublin, Dublin D02 DK07, Republic of Ireland; Academic Unit of Neurology, Trinity College Dublin, Trinity Biomedical Sciences Institute, Dublin D02 PN40, Republic of Ireland; Department of Neurology, Beaumont Hospital, Dublin D02 PN40, Republic of Ireland; Department of Basic and Clinical Neuroscience, King’s College London, Maurice Wohl Clinical Neuroscience Institute, London SE5 9RS, UK; Department of Neurology, King’s College Hospital, Bessemer Road, London, SE5 9RX, UK; Department of Neurology, UMC Utrecht Brain Center, 3584 CG, Utrecht, the Netherlands; University of Exeter Medical School, University of Exeter, Exeter EX2 5DW, UK; Department of Neurology, UMC Utrecht Brain Center, 3584 CG, Utrecht, the Netherlands

## Abstract

Illumina DNA methylation arrays are a widely used tool for performing genome-wide DNA methylation analyses. However, measurements obtained from these arrays may be affected by technical artefacts that result in spurious associations if left unchecked. Cross-reactivity represents one of the major challenges, meaning that probes may map to multiple regions in the genome. Although several studies have reported on this issue, few studies have empirically examined the impact of cross-reactivity in an epigenome-wide association study (EWAS). In this paper, we report on cross-reactivity issues that we discovered in a large EWAS on the presence of the *C9orf72* repeat expansion in ALS patients. Specifically, we found that that the majority of the significant probes inadvertently cross-hybridized to the *C9orf72* locus. Importantly, these probes were not flagged as cross-reactive in previous studies, leading to novel insights into the extent to which cross-reactivity can impact EWAS. Our findings are particularly relevant for epigenetic studies into diseases associated with repeat expansions and other types of structural variation. More generally however, considering that most spurious associations were not excluded based on pre-defined sets of cross-reactive probes, we believe that the presented data-driven flag and consider approach is relevant for any type of EWAS.

## INTRODUCTION

DNA methylation is a key epigenetic mechanism that is involved in gene regulation by influencing transcription factor binding and recruiting histone-modifying proteins (1). It involves the addition of a methyl group to the DNA, which occurs predominantly at CpG dinucleotides. DNA methylation patterns are propagated through cell division and play a key role in development, where it is involved in tissue-specific transcriptional regulation and genomic stability (2). DNA methylation at certain loci remains dynamic throughout life and can be influenced by the environment, lifestyle and ageing (3). Importantly, aberrations in DNA methylation patterns have been associated with a wide range of human diseases including cancer, cardiovascular disease and schizophrenia (3–5).

An important driver in expanding our understanding of DNA methylation in health and disease has been the growing number of epigenome-wide association studies (EWAS) (6). These studies were facilitated by the advance of high-throughput techniques that quantify DNA methylation at sites across the genome. Among these techniques, the Illumina Infinium BeadChip arrays have been used most widely, offering genome-wide coverage at a relatively low cost (7). There are several generations of Illumina methylation beadchips available (27K, 450K and EPIC array, respectively), which all use similar probe-based technology, but newer generations see increased coverage. The Infinium methylation technology is based on bisulfite treatment of DNA, which converts all unmethylated Cs into Ts, thereby introducing a C/T genetic variant in CpG-sites that can be interrogated using microarray technology.

Although several studies have shown that these arrays generally provide accurate and reproducible measures, there have been various reports on technical artefacts that can result in spurious results (8–12). Cross-reactivity presents one of the major technical artefacts, where probes may map to multiple locations in the genome and therefore measure a mixture of specific and aspecific signals. In the first report on cross-reactivity in Illumina DNA methylation arrays the authors showed that many sex-associated autosomal probes on the 27K array were caused by cross-hybridization to the sex chromosomes (13). Since then, several studies have reported on cross-reactive probes in both the 450k and EPIC array, resulting in a variety of probes that should be excluded (9–12). More often than not, this concerns a substantial number of probes (ranging from 6 to 11% of all probes). However, despite these comprehensive efforts, there is still a lack of studies showing the actual impact of cross-hybridization in EWAS.

Here, we report on unreported issues of cross-reactivity that we discovered while pursuing EWAS in large cohorts of patients with amyotrophic lateral sclerosis (ALS) and controls. Specifically, we focused on the identification of DNA methylation profiles associated with the presence of the *C9orf72* (C9) repeat expansion, a GGGGCC (G_4_C_2_) nucleotide repeat expansion, which is the most common mutation (±8%) in both ALS and frontotemporal dementia (14). We provide compelling evidence that the majority of the loci that were associated with the presence of the C9 repeat expansion result from cross-hybridiziation to the repeat sequence. Importantly, these issues affected the majority of the significant results, and the spurious associations were not excluded based on sets of cross-reactive probes established in previous studies. In this paper we show that: (i) limited (≤30 bp) off-target sequence matches can result in cross-hybridization in Illumina Methylation arrays, which is below the criteria used in previous research; (ii) imperfect matches (i.e. with mismatches/INDELs) to off-target regions can result in spurious associations; and (iii) genetic variation (especially tandem repeats and other types of structural variation) associated with the phenotype of interest could severely confound EWAS analyses because they are not included in existing annotations of cross-reactive probes that are based on the reference genome.

We show that these issues apply to both the most recent EPIC array as well as the older, but more widely used, 450k array. Our findings serve as a cautionary note to researchers using Illumina arrays, and we provide several recommendations to prevent spurious results caused by cross-hybridization.

## MATERIALS AND METHODS

### Study population

All ALS patients included in this study were collected as cohorts in Project MinE (www.projectmine.com), described in more detail elsewhere (15).

#### NL

All participants gave written informed consent and approval was obtained from the local, relevant IRB committees for medical research. DNA methylation profiling was performed on 2916 samples, comprising 1867 patients with ALS and 1049 controls. All patients were diagnosed according to the revised El Escorial criteria. Control subjects were from ongoing population-based studies on risk factors in ALS.

#### UK

Cases were diagnosed with probable or definite ALS according to the 1994 El Escorial Criteria by neurologists specialized in motor neuron diseases. Control samples were collected from neurologically normal, unrelated individuals, either spouses of ALS patients, carers or blood donors from the same geographical region. DNA methylation profiling was performed on 383 samples, comprising 266 patients with ALS and 117 controls.

#### Ireland

Cases were diagnosed with probable or definite ALS according to the 1994 El Escorial Criteria by neurologists specialized in motor neuron diseases Beaumont Hospital in Dublin. Patients were referred from all regions in Ireland and were part of an ongoing population-based prospective ALS registry. Control samples were matched for gender and age. They were either spouses or those accompanying patients to the ALS clinic. All individuals reported Irish ancestry for at least three generations. DNA methylation profiling was performed on 298 samples, comprising 200 patients with ALS and 98 controls.

### DNA methylation profiling

Venous blood was drawn from patients and controls from which genomic DNA was isolated using standard methods. We set the DNA concentrations at 100 ng/μl as measured by a fluorometer with the PicoGreen^®^; dsDNA quantitation assay. DNA integrity was assessed using gel electrophoresis. Genomic DNA (∼1 μg) was bisulfite-treated using Zymo Bisulfite Conversion Kits (Zymo Research, Orange, CA, USA). DNA methylation was analyzed using the Infinium Methylation450k array (NL samples) or Infinium EPIC array (UK and Ireland samples), according to the standard Infinium HD array methylation protocol (Illumina, San Diego, CA, USA)

### Software & Availability

For EWAS analyses we used the OSCA software v0.41 (available at: http://cnsgenomics.com/software/osca) (16). All other analyses were performed in the statistical programming language R (version 3.5) (17). Figures were made with the R package *ggplot2*, using the colorblind-friendly color palette published by Ichihara *et al.* (18,19). Figure 2A was made using the *pheatmap* package (20). Probe mapping was performed using the *Biostrings* package (21). Finally, several other packages were used, mostly from the *tidyverse* (22), for a complete overview of packages see the github page below. All code is available at https://github.com/pjhop/dnamarray_crossreactivity which includes documentation on the data scructure and scripts used. Furthermore, we made several main functions are available as an installable R package at: https://github.com/pjhop/DNAmCrosshyb.

### Quality control & normalization

Raw signal intensities were read into R using the *minfi* package (23).

#### Sample QC

We performed quality control and normalization separately for the 450k data (NL) and EPIC data (UK and Ireland). Identical thresholds were used for 450k and EPIC data, unless indicated. Samples that failed the following criteria were removed: (i) Samples with median methylated or unmethylated intensity <1500 (<2000 for EPIC data). (ii) Samples with median red/green ratio <0.5 or >2 as calculated in type I probes (<0.4 or >2.5 in EPIC data). (iii) Discordance between reported sex and predicted sex based on the *getSex* function in *minfi* (23). (iv) Samples that failed on the OP (non-polymorphic controls) or Hyb (hybridization controls) metrics as implemented in the *methylaid* package (24). (v) Samples with incomplete bisulfite conversion (<80%) based on the *bscon* metric as implemented in the *wateRmelon* package (25). (vi) Samples where >5% of probes had detection *P*-value < 1 × 10^−16^ and/or >5% of probes were measured by <3 beads. (vii) Samples that failed on the inbreeding and relatedness metrics in the corresponding whole-genome-sequencing (WGS) data. Quality control of the Project MinE WGS data was performed as described earlier (15). (viii) We removed samples that did not match their respective genotype data. Briefly, we used the *omicsPrint* package to select 200 probes that reliably measured underlying SNPs and were present in the WGS-derived SNP data (26). We performed identity-by-state (IBS) between the DNAm-inferred SNPs and the WGS-derived SNP data using the *allelesharing* function. We removed samples for which the DNAm-inferred SNPs did not match the WGS-derived SNPs (IBS mean <1.9 and/or IBS variance >0.1). Finally, we removed one individual for each related pair of individuals (identical or first-degree) to obtain a set of unrelated individuals. The number of samples that failed on the various QC metrics are listed in Supplementary Tables S1 and 2. The baseline characteristics for samples that pass QC are provided in Supplementary Tables S3 and 4.

#### Probe QC

We first removed samples that failed QC and then set all the measurements with detection *P*-value > 1 × 10^−16^ or measured by <3 beads to missing (27). We then removed all probes with >5% missing data.

#### Normalization

The QC’ed signal intensities were normalized using the *dasen* function as implemented in the *wateRmelon* package (25). For type I probes, we also extracted the out-of-band (OOB) signal intensities (28) (see next section). The OOB signal intensities were normalized using the *naten* function as implemented in the *wateRmelon* package (25). Note that *naten* performs the same normalization procedure as *dasen*, except that it does not equalize type I and type II backgrounds (which is not relevant for OOB intensities, since these exist only for type I probes).

### Out-of-band (OOB) signal

Color channel switches can occur in type I probes, where signals from both the methylated and unmethylated beads are measured in the same designated color channel. The unused color channel is termed the OOB channel. If a probe hybridizes to an off-target region, the base preceding the off-target CpG-site may be different than the base preceding the intended CpG-site. If so, this can result in the incorporation of a differently labeled nucleotide, in which case signal from the off-target region would be measured in the OOB color channel.

### Calculation of in-band and OOB β-values

We transformed the normalized signal intensities into β-values, using the following formula:

(1) \begin{equation*} \beta =\frac{M}{M + U + 100} \end{equation*}

Here, *M* represents methylated intensity and *U* represents unmethylated intensity. We used the same formula for in-band and OOB signal intensities.

### Phenotypes

*C9orf72* expansion status was determined using the Illumina ExpansionHunter tool where subjects with ≥30 repeats were classified as carriers of the repeat expansion (29,30). Since chronological age, smoking status and white blood cell (WBC) fractions were not available for all samples, we used established prediction algorithms to impute them. Age was predicted using the *agep* function in the *wateRmelon* package, which uses the coefficients from Horvath’s multi-tissue age predictor (25,31). We calculated a smoking score as previously described in Elliot *et al.* and implemented in the *EpiSmoker* package (32,33). We imputed white blood cell fractions (CD8T cells, CD4T cells, Monocytes, Granulocytes, B cells and NK cells) using the *EpiDish* package, where we used the ‘RPC’ (Robust Partial Correlations) algorithm (34). Since the WBC fractions always add up to one, we dropped one cell-type (B-cells) in the analyses to prevent multicollinearity among the WBC covariates.

### EWAS on *C9orf72* expansion status

We performed an EWAS of *C9orf72* expansion status within ALS cases using a mixed linear model as implemented in the OSCA software (16). Briefly, this algorithm tests for an association between the methylation status of a CpG-site (β-value) and a trait (in this case *C9orf72* status) while fitting all the other distal probes as random effects. Fitting the distal probes as random effects accounts for correlations induced by (unobserved) confounding factors. Specifically, we used the ‘LOCO’ option, which excludes all probes located on the same chromosome as the target site from the random effects terms so that the target site is not fitted twice (once as a fixed effect and once as a random effect). We included sex, experimental batch, predicted age and predicted smoking score as fixed covariates in the model.

We used the same algorithms as described above for the EWASs on OOB β-values and EWASs on total signal intensities. In case the OSCA algorithm did not converge, we used a multivariate linear model adjusting for experimental batch, age, sex, smoking score, imputed cell fractions, the first five array-wide PCs and the first five control probe PCs. This was the case for the EWAS on total signal intensities and the EWAS in the EPIC replication dataset.

### Identification of probes that (partially) map to the C9 repeat

We generated the forward and reverse strand of the GGGGCC hexanucleotide repeat *in silico*. Although the number of repeats differs per carrier, we used a fixed number of 10 repeats (60 bp), which is sufficient for the full 50-nt sequence of a probe to match. Both strands were bisulfite-converted *in silico*, where all non-CpG cytosines were converted in T’s and C’s in CpG-sites were either converted to Ts (unmethylated) or Cs (methylated). We then generated the complement strands of the bisulfite-converted strands, resulting in four strands in total (forward, reverse, forward complement and reverse complement).

We downloaded the Illumina 450k and EPIC annotations as implemented in Bioconductor (35). The probe sequences for type I probes were directly extracted from the annotation files, consisting of two probe sequences per CpG-site (methylated + unmethylated). Type II probes contain up to three R bases (R = A or G in IUPAC code) in the probe sequence, resulting in up to 8 (2^3^) possible probe sequences per CpG-site. We generated all possible combinations for the type II probes and combined them with the type I probe sequences, resulting in 1 119 157 450k probe sequences and 1 752 933 EPIC probe sequences.

For each width between 1 and 50 bp we scanned the 3′-subsequence of the probe’s sequence for overlap with the four bisulfite-converted hexanucleotide repeat strands using the *Biostrings* package in R (21). We repeated this procedure for several scenarios:

- Assuming that the repeat is either fully methylated (all C’s of CpGs remain C’s), or that the repeat is fully unmethylated (all C’s of CpGs are converted into T’s).
- Allowing every combination of DNA methylation states within the repeat by converting all C’s of CpGs to Y’s (Y = T or C in IUPAC code).
- Allowing a mismatch or an INDEL >5 bp of the 3′end of the probe sequence. The 5 bp cutoff is based on the finding by Zhou *et al.* that variation nearer to the 3′ site of the probe will not result in hybridization (12).

## RESULTS

### EWAS on *C9orf72* status identifies 18 genome-wide significant loci

**Figure 1.**
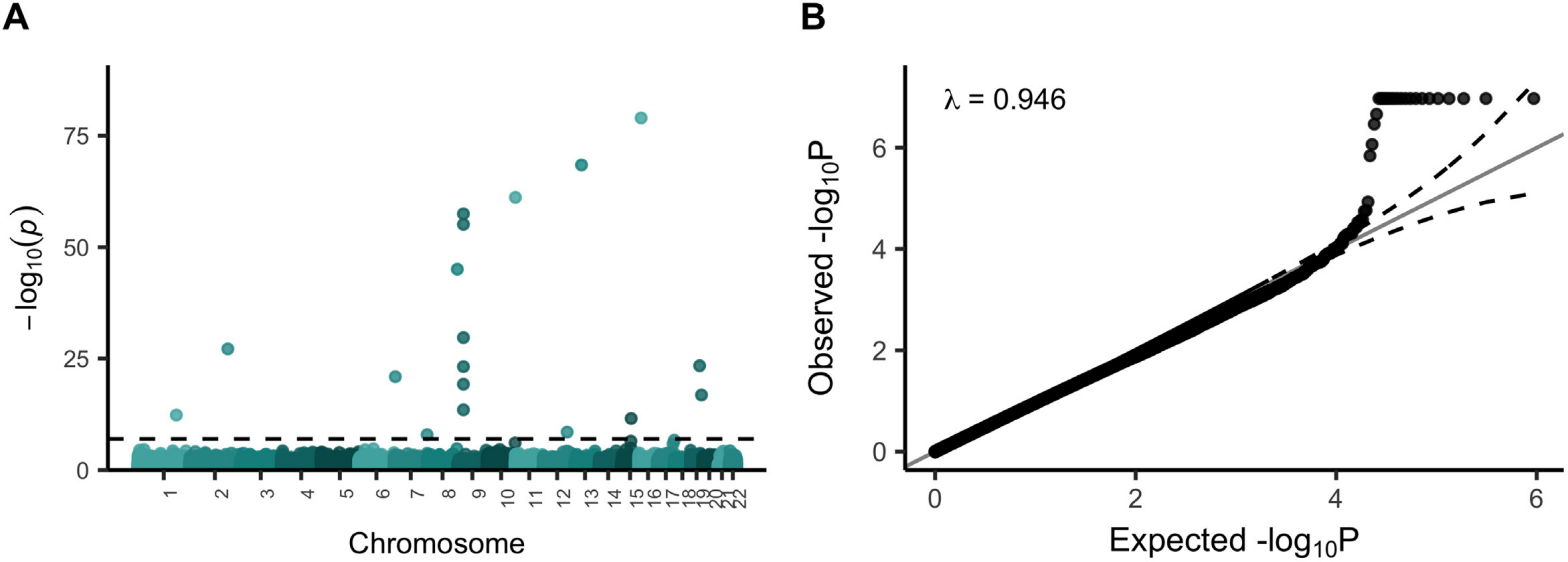
EWAS on C9 status within ALS patients. (**A**) Manhattan plot comparing association *P*-values (−log_10_(*P*), *y*-axis) and genomic location (*x*-axis). The dashed line indicates the bonferroni significance threshold (1.1 × 10^−7^). (**B**) QQ-plot showing observed *P*-values (−log_10_(*P*), *y*-axis) against the expected distribution under the null (*x*-axis). For presentation purposes *P*-values <1.1 × 10^−7^ are plotted as 1.1 × 10^−7^.

After stringent quality control, we performed an EWAS of *C9orf72* (C9) repeat expansion status (wild-type or expanded) within 1748 ALS patients (see Supplementary Tables S1–3 for an overview of QC steps and study population). We tested for an association between DNA methylation at 467 303 sites (450k array) and C9 status, using mixed linear models where all distal probes were fitted as random effects as implemented in the OSCA software (16). Specifically, we used the LOCO (leave-one-chromosome-out) option to prevent the same probe being fitted twice and included age, sex, smoking score and experimental batch as fixed covariates. We observed modest deflation of test-statistics in the quantile–quantile plot (*λ* = 0.946, Figure 1B), and sensitivity analyses indicated that the results were robust to changes in analysis strategy (Supplementary Figure S1). We identified 18 sites at which DNA methylation was significantly associated with the presence of the C9 repeat expansion at a Bonferroni-corrected significance threshold (*P* < 1.1 × 10^−7^, Figure 1B; Supplementary Table S10). Six of the significant CpG-sites were located *in cis* of the C9 repeat (<100 kb), with four of these located in a CpG island directly upstream of the repeat. The remaining 12 CpGs were located *in trans* of the C9 repeat expansion, being distributed across chromosomes (Figure 1A). All significant *trans* sites showed increased DNA methylation levels in C9 carriers.

### Significant *trans* CpG-sites exhibit ambiguous characteristics

**Figure 2.**
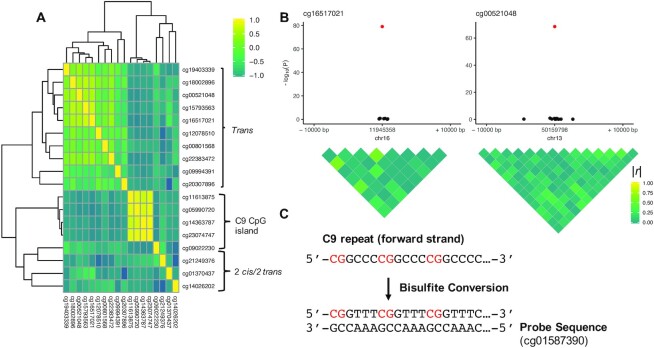
(**A**) Heatmap showing the correlations between the 18 CpGs that were significantly associated with C9 status. Correlations were calculated within carriers of the C9 expansion. Probes are annotated with their respective positions: the upper cluster consists of 10 probes *in trans* (>100 kb) of the C9 repeat, the middle cluster consists of four probes located in the CpG island upstream of the repeat, and the lower cluster consists of two *trans* probes and two *cis* probes. (**B**) Locus plots of the two most significant *trans* CpGs. There are no regional effects in the *trans* associations. Locus plots for all *trans* probes are shown in Supplementary Figure S2. (**C**) Example of the C9 GGCCCC hexanucleotide repeat after bisulfite conversion (forward strand shown), where we assumed that the repeat is fully methylated (43). The probe sequence of one of the *trans* CpG-sites (cg01587390) is displayed, showing that the 3′ end of the probe partially matches the bisulfite-converted C9 repeat.

Although the identified *trans* CpG-sites initially seemed interesting, several observations led us to suspect that these associations actually reflected technical artefacts. First, we observed notable correlations in DNA methylation levels across the majority of *trans* CpG-sites (Figure 2A), suggesting that a common factor underlies these associations. Although this might reflect a coordinated DNA methylation signal, it might also indicate that a common technical or biological confounder is influencing DNA methylation levels across sites (16). Second, closer inspection of the *trans* loci showed that the associations did not extend to the regions surrounding the *trans* CpG-sites (Figure 2B and Supplementary Figure S2). Importantly, technical artefacts are more likely to affect single CpGs (36). Inspection of the specific probe sequences revealed that 11 (92%) of the *trans* probes share a similar 3′ sequence (Supplementary Table S5), with the 3′-ends of these probes showing homology to the bisulfite-converted C9 repeat expansion (note that each repeat contains one CpG-site, Figure 2C).

Together, these observations suggest that the identified *trans* associations do not reflect true DNA methylation differences at the intended regions, but instead reflect differential DNA methylation at the C9 repeat due to cross-hybridization. Since the size of the C9 expansion ranges from hundreds to thousands of repeats, a sequence match to the repeat sequence could lead to many off-target hybridization events in carriers of the mutation. This would in turn lead to an increased signal intensity for these probes, and importantly, could lead to shifts in β-values resulting in differential methylation readouts.

In the following sections we provide a more detailed examination of this issue, leading to novel insights regarding cross-hybridization in Illumina methylation arrays.

### Identification of probes that map to the C9 repeat

To determine the extent of probes that (partially) match the C9 repeat, we mapped all probes on the 450k array (485 512 probes) to the bisulfite-converted C9 repeat sequence *in silico*. Briefly, for each width between 1 and 50 bp we scanned the 3′-subsequence of the probe sequence for a match with the four bisulfite-converted hexanucleotide repeat strands. We first focused on type I probes (since all suspicious *trans* probes were this bead type) and assumed that the repeat was fully methylated (all CpGs in the repeat are assumed to be methylated, Figure 2C).

**Figure 3.**
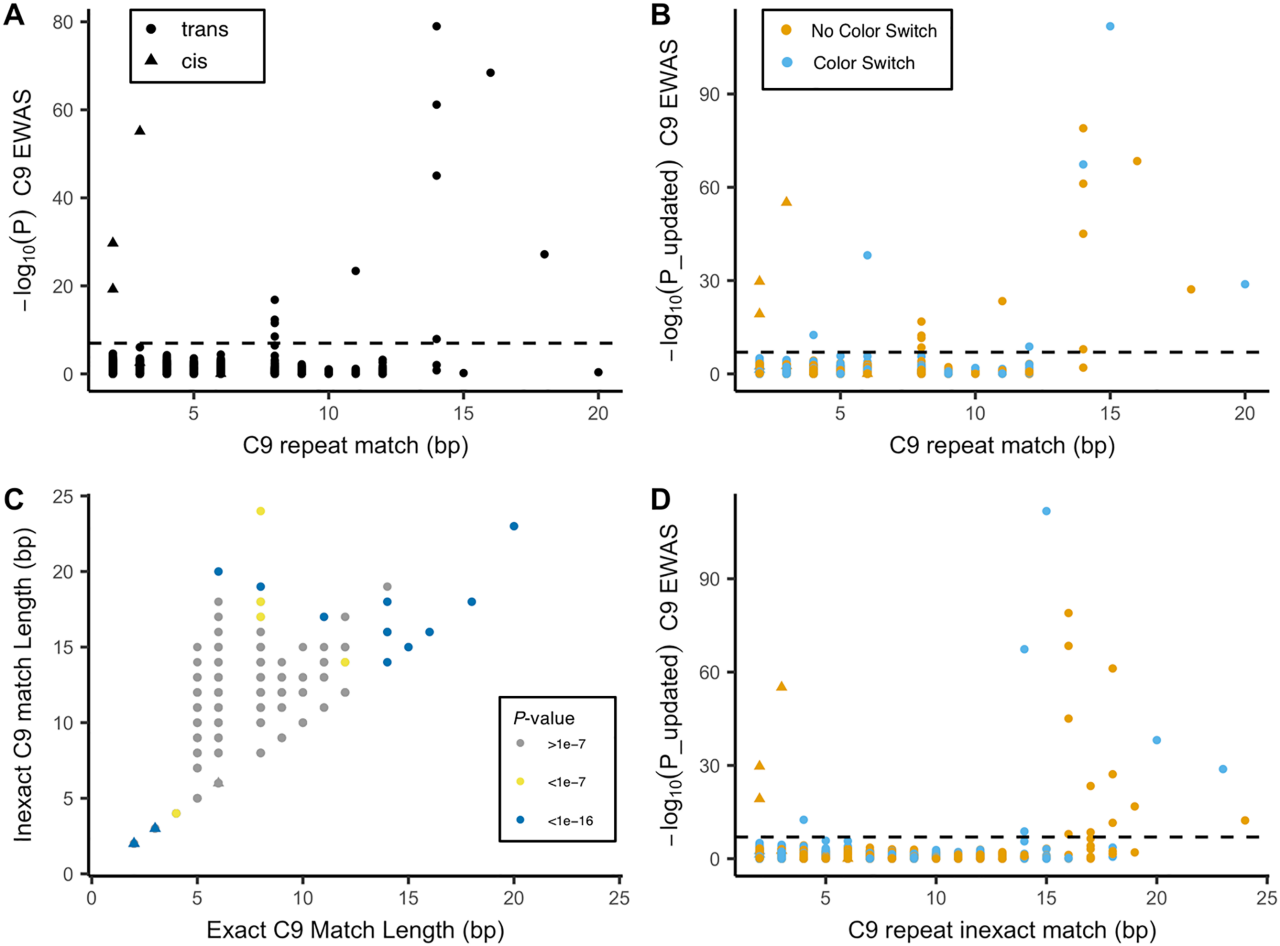
Comparison between *P*-values from the *C9orf72* EWAS and match (bp) between the probe’s 3′-subsequence and the bisulfite-converted *C9orf72* repeat (see Supplementary Figure S4 for type II probes). Here, we assumed that the repeat was completely methylated (i.e. all Cs converted to Ts, except C’s in CpG-sites). (**A**) Comparison between *P*-values from the C9 EWAS and match (bp) between the probe’s 3′-subsequence and the bisulfite-converted C9 repeat. (**B**) Comparison between the *P*-values (−log_10_(*P*), *y*-axis) from the C9 EWAS and match between the probe’s 3′-subsequence. *P*-values from the EWAS on OOB β-values were used in case of a predicted color channel switch (OOB probes). (**C**) Comparison between the length of the inexact match to the C9 repeat (*y*-axis) and exact match to the C9 repeat for type I probe sequences. Points are colored by significance levels in the C9 EWAS. (**D**) Comparison between the *P*-values (−log_10_(*P*), *y*-axis) from the C9 EWAS and inexact match between the probe’s 3′-subsequence. *P*-values from the EWAS on OOB β-values were used in case of a predicted color channel switch (OOB probes).

As Figure 3 A shows, several significant *trans* probes partially match (≥14 bp) the C9 repeat, while none of the probe sequences of the significant *cis* associations match the repeat (note that we used cutoff of ≥14 bp to define probes that partially match to the C9 repeat, we will consider this cutoff in more detail later). We found however that, some probes that partially match to the C9 repeat failed to reach significance in the EWAS. Moreover, we note that several genome-wide significant *trans* probes had only a limited match to the C9 repeat (>5 bp, <14 bp). We discovered that these observations could be explained by color channel switches and imperfect matches respectively, which we discuss in more detail in the following section.

#### Color channel switches

We hypothesized that the lack of association of some of the *trans* probes that *do* partially match the C9 repeat could be explained by color channel switches (4 out of 10 probes with a ≥14 bp C9 match were not significant). To investigate this hypothesis we first predicted for each probe whether a color-channel switch would occur if the probe would hybridize to the C9 repeat (see Materials and Methods section). For probes with an expected color-channel switch upon C9 hybridization (dubbed ’OOB probes’), we expect differential signal in the OOB color channels. We therefore performed an EWAS using β-values calculated from the OOB channels (dubbed OOB EWAS, Supplementary Figure S3). Nine probes were significant in the OOB EWAS, of which six were were predicted to result in OOB signal upon hybridization to the C9 repeat (OOB probes). The remaining 3 sites were significant in both the original EWAS and the OOB EWAS.

In Figure 3B we highlight the OOB probes and updated the *P*-values of these probes with *P*-values from the OOB EWAS (Supplementary Figure S3). Strikingly, the OOB probes with a ≥14 bp match that were not significant in the original EWAS (three probes), *were* highly significant in the OOB EWAS. In total, using β-values calculated in the predicted color channels, 9 out of 10 type I probes with a ≥14bp C9 match were significantly associated with C9 status.

#### Inexact matching

We hypothesized that the significant *trans* probes with a limited C9 repeat match (six in-band probes and three OOB probes) may match imperfectly (i.e. with mismatches and/or gaps). We therefore reran the mapping procedure using inexact matching where we allowed one mismatch/INDEL. We excluded matches with a mismatch/INDEL near the 3′-end of the probe (≤5 bp) since it has been previously shown that these prevent hybridization (12). Several probes showed a markedly increased match length when inexact matching was applied (Figure 3C). Seven out of the nine significant *trans* probes with a <14 bp exact C9 match had an ≥14 bp inexact C9 match. This suggests that an inexact match between a probe and the DNA sequence can lead to sufficient off-target hybridization to result in spurious associations.

We repeated the above analyses for type II probes, finding that relatively fewer type II probes partially match the C9 repeat: one probe had an exact match of ≥14 bp, and this number increased to 30 probes when applying inexact matching (Supplementary Figure S4). There were no significant associations among these probes.

### Majority of results are affected by previously unidentified cross-hybridization issues

#### Enrichment of C9-mapping probes

In total, we identified 137 probes with an ≥14 bp inexact match to the C9 repeat (dubbed the C9-mapping probes). These include the majority of the sites that were significantly associated with C9 status (11 out of 18, Supplementary Figure S5A), representing a strong enrichment (OR = 5288, *P* =1.9 × 10^−35^, Table 1). We further found that eight out of the nine significant sites identified in the OOB EWAS were among the C9-mapping probes (Supplementary Figure S5B and Table 1), providing strong evidence that cross-hybridization can lead to detectable OOB signal.

**Table 1.** Number of significant sites in the EWAS and the OOB that were identified as mapping to the C9 repeat

	Annotated location (*cis*/*trans* of C9 repeat)	N significant probes	C9-mapping cross-reactive probes*	C9-mapping probes across varying mapping strategies**
EWAS	*cis*	6	0 (0%)	0 (0%)
	*trans*	12	11 (92%)	11 (92%)
OOB EWAS	*cis*	0	0 (0%)	0 (0%)
	*trans*	9	8 (89%)	9 (100%)

*Defined as an ≥14 bp inexact match to the C9 repeat (allowing one mismatch/INDEL >5 bp from the 3′-end of the probe).

**See Supplementary text and Supplementary Figures S10–15. One significant OOB site was classified as C9-mapping when allowing mismatch/INDELS closer to the 3′-end of the probe.

We do note that the chosen 14 bp cutoff used to define C9-mapping probes is data-driven and could therefore be biased (the cutoff was based on the observation that most significant *trans* probes had a ≥14 bp match to the C9 repeat). However, we found that for a range of cut-offs, the significant probes from the C9 EWAS were strongly enriched for probes with longer matches to the C9 repeat (Supplementary Figure S6). We note that we made several assumptions in matching the probes with the C9 repeat expansion. We therefore performed several sensitivity analyses that showed that different assumptions have little impact on the results (see Supplementary Figure S10 and the Supplementary text). We did find, however, that allowing a mismatch/INDEL at any position in the probe (instead of >5bp from the 3′-end) led to an additional significant OOB probe being classified as C9-mapping (≥14 bp C9 match), resulting in all 9 significant OOB probes being classified as C9-mapping (Table 1). This indicates that off-target matches with a mismatch close to the 3′-end of the probe may still result in cross-hybridization. Finally, we assessed whether the identified associations were driven by outliers, which could indicate that a few carriers with very large repeat expansions drive the results. We found however, that there were marked differences in median β-values for both the in-band and OOB C9-mapping probes (Supplementary Figures S8 and 9), suggesting that the identified associations were not driven by outliers.

#### Evaluation of existing methods

Previous studies on cross-hybridization have reported varying numbers of probes that may hybridize to multiple locations in the genome (10–12). However, we found that few to none of the cross-hybridizing probes identified in this study were flagged as such in previous studies (Supplementary Table S6). Moreover, we evaluated several methods designed to adjust for hidden confounders in EWAS studies and found that, regardless of the method used, 40–60% of significant results were affected by cross-hybridization (Supplementary Table S7) (16,37–39). Together, these findings suggest that existing methods to account for bad quality probes and confounding may not suffice in preventing spurious results due to cross-hybridization.

### C9-mapping probes show significant differences in signal intensity

**Figure 4.**
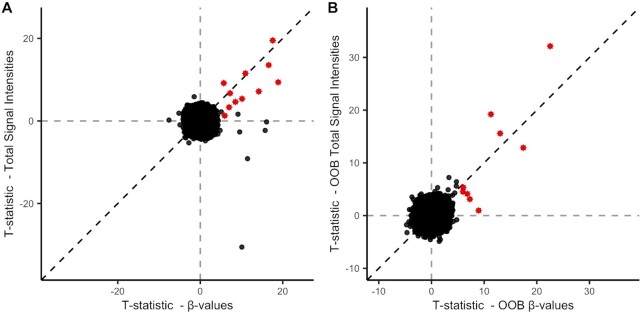
Comparison between association test-statistics between C9 status and total signal intensities (*y*-axis) and association test-statistics between C9 status and β-values. (**A**) In-band test-statistics. Probes flagged as mapping to the C9 repeat (≥14 bp inexact match) and that were significant in the EWAS are highlighted (fourth column in Table 1). (**B**) OOB test-statistics. Probes flagged as mapping to the C9 repeat (≥14 bp inexact match) and that were significant in the OOB EWAS are highlighted (fourth column in Table 1).

To further substantiate that the observed findings are caused by cross-hybridization, we evaluated the total signal intensities at these sites. For probes that map to the C9 repeat, we expect more hybridization events in carriers of the repeat expansion, which would in turn lead to an increased total signal intensity. To test this, we tested for an association between total signal intensity (*M* + *U*) and C9 status at each probe, using a mixed linear model as implemented in OSCA. We identified 11 probes for which the total signal intensity was significantly associated with C9 status (*P* < 1.1 × 10^−7^, Figure 4A). The majority of significant probes (8 out of 11) were C9-mapping probes that were also significant in the EWAS on β-values (Figure 4A) (Of note, three sites that showed diminished total intensities in C9 carriers (right lower quadrant in Figure 4A) were located in the immediate vicinity of the C9 repeat, presumably because of frequent deletions that have been reported previously (40,41)). All intensity-associated C9-mapping probes showed higher intensities in carriers of the expansion, indicating more hybridization events at these probes in the carriers. We further found that the total OOB signal intensities of nine probes were significantly associated with C9 status, all being C9-mapping probes of which most were significant in the OOB EWAS (Figure 4B). Finally, we found that these results could not be explained by confounding signal saturation effects (see Supplementary results for details).

### The EPIC array shows similar cross-hybridization issues

**Figure 5.**
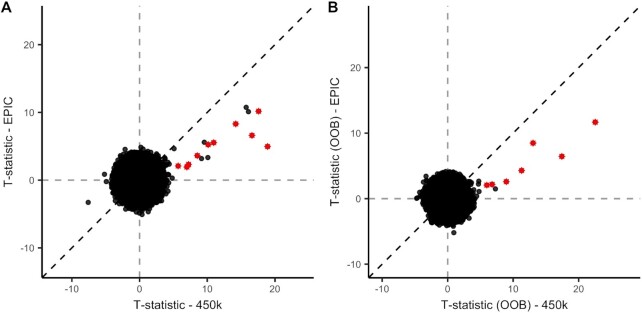
Comparison between test-statistics from the EWAS in the 450k cohort and test-statistics from the EWAS in the EPIC cohort. Probes flagged as mapping to the C9 repeat (≥14 bp inexact match) and that were significant in the 450k EWAS are highlighted. (**A**) T-statistics from the EWAS in the EPIC cohort (y-axis) compared with t-statistics from the EWAS in the 450k cohort (*x*-axis). (**B**) T-statistics from the EWAS on OOB β-values in the EPIC cohort (*y*-axis) compared with t-statistics from the EWAS on OOB β-values in the 450k cohort (*x*-axis).

We performed a replication analysis in two cohorts comprising 437 ALS patients that were profiled using the more recent Illumina EPIC array (Supplementary Table S4). This way we not only assess whether the cross-hybridization issue is specific to our experiment, but also whether this issue persists on the EPIC array. Out of the 18 significant loci identified in the 450k data, 16 were present in the EPIC data. Thirteen out of 16 loci replicated in the EPIC data at a replication threshold (*P* = 0.05/16 = 0.003), and had a consistent direction of effect (Figure 5 A and Supplementary Figure S17). The replicated loci included seven *trans* probes that had a ≥14 bp match to the C9 repeat. Similarly, four out of the nine probes that were significant in the 450k OOB EWAS replicated in the EPIC data (Figure 5B). To evaluate whether the EPIC array contains additional probes that map to the C9 repeat, we mapped all EPIC probes to the bisulfite-converted C9 repeat. We found that the EPIC array contains 1127 C9-mapping probes (≥14bp match to either unmethylated or methylated C9 repeat), of which 342 were specific to the EPIC array. DNA methylation at three of these EPIC-specific probes were significantly associated with carrier status (*P* < 0.05/1127). Finally, similar to the 450k array, we found that the significant probes from the C9 EWAS in the EPIC data were enriched for probes with longer matches to the C9 repeat (OR = 333, *P* = 2.3 × 10^−9^).

## DISCUSSION

In this paper, we report on hitherto undiscovered cross-hybridization issues in Illumina DNA methylation arrays. We discovered these issues in a large EWAS on the presence of the *C9orf72* (C9) repeat expansion in ALS patients. We provide strong evidence that the majority of the significant associations were spurious due to cross-hybridization to the C9 repeat. Our findings highlight the extent to which cross-reactivity can impact EWAS findings. Although previous studies have reported on cross-reactivity in Illumina methylation arrays, only 2 out of 11 of the technical artefacts would have been removed based on existing guidelines. These findings are particularly relevant for epigenetic studies into diseases associated with repeat expansions and other types of structural variation. More generally however, we believe that the data-driven flag&consider approach we employ in this study is relevant for any type of EWAS, since we show that removing pre-defined sets of probes may miss spurious associations.

We found several convergent lines of evidence that strongly suggest that the majority of significant CpG-sites were false positives due to cross-hybridization to the C9 repeat expansion. First, among the significant sites we found a strong enrichment for probes with longer sequence matches to the C9 repeat. This enrichment was present in both the 450k and EPIC array data. Second, we show that probes predicted to cause fluorescent signal in the (unintended) OOB color channel upon cross-hybridization to the C9 repeat indeed showed differential signal in these channels. These findings indicate a novel use of the OOB signals, and adds to previous studies that have shown that OOB signals can provide valuable information (28,42). Third, the probes that partially match the C9 repeat showed increased signal intensity levels in carriers of the expansion. This indicates that these probes measure copy number differences between carriers and non-carriers, which supports the hypothesis that these probes hybridize to the C9 repeat. Finally, these probes were strongly correlated—albeit being spread across the genome—and their direction of effect was consistent with those found in the C9 repeat in previous studies (43).

Spurious associations due to cross-hybridization have long been recognized and various probe filtering approaches have been proposed to prevent them (9–12). The most widely used method involves removing probes with a ≥47 bp match to an off-target region (10). In addition, it has been proposed to remove all probes that overlap with repetitive regions (11). More recently, a data-driven approach was used to show that probes with off-target matches of 30 bp or greater can lead to cross-reactive signals (12).

In this study, we made several novel observations that explain why the issues we discovered were not identified in any of the aforementioned studies. Specifically, we show that genetic variation should be taken into account when considering potential cross-hybridization issues, since these are not covered by existing methods that map probes to the reference genome. This is especially relevant when the phenotype being studied is associated with repeat expansions or other types of structural variation, which have been implicated in a substantial number of diseases (44). To exemplify this, we show in Supplementary Tables S8 and 9 that similar issues may be expected in diseases associated with other types of repeat expansions. Although these findings are particularly relevant for the cases described above, our findings also have more general implications. First, our results suggest that off-target sequence matches below the recently proposed 30 bp cutoff can lead to spurious associations (12). Moreover, we found that imperfect off-target matches (i.e. allowing for mismatches/INDELs) can impact β-value readouts. In addition, inexact matching implicitly takes into account that a probe may have an off-target match in some individuals and not in others due to genetic variation (i.e. a probe may only match an off-target region when an individual carries a variant, which is not taken into account by mapping to the reference genome).

An issue that emerges from this study is that off-target sequence matches as low as 14 bp resulted in spurious associations. Excluding probes based on the aforementioned cutoff would lead to the removal of practically all probes, and is therefore not a sensible strategy. Whether off-target matches lead to detectable shifts in β-values will ultimately depend on several factors. These include the combination of the number of off-target matches and the lengths of these matches. Whereas one or a few long off-target matches may be sufficient for detectable cross-hybridization, a larger number of short matches will generally be required to reach detectable levels of off-target signal. Moreover, correlated off-target signals will shift β-values in the same direction and are thus more likely to cause detectable shifts. In this regard, we expect that many copies of an off-target sequence are required before matches as small as those described here (≥14 bp) will result in spurious associations. In addition, other factors may influence the likelihood of cross-hybridization, such as the experimental washing protocols and GC-content of the probe (45,46).

Due to this combination of contributing factors, it is not straightforward to decide *a priori* which probes should be excluded, and relying on a fixed threshold to exclude cross-reactive probes can miss spurious associations. This conclusion is supported by the fact that most of the cross-reactive probes we discovered were not flagged as such in previous studies. Moreover, we note that these phenomena, although unintended, may represent biological signals of interest. We therefore argue for a ‘flag and consider’ approach rather than removing specified sets of probes beforehand, as previously suggested regarding probes that overlap with genetic variation (47).

Several analytical checks used in this study can aid in identifying probes that may be affected by cross-hybridization. First, probes identified in an EWAS should be checked for off-target sequence matches. In contrast to previous studies, we recommend considering off-target matches <30 bp and allowing for imperfect matches. In addition, any known genetic variation associated with the phenotype should be taken into account. We made scripts to map significant probes to both reference and non-reference sequences available in an R package (https://github.com/pjhop/DNAmCrosshyb). Second, we recommend inspecting the results of an EWAS for the presence of various ’red flags’, which can point toward cross-hybridization issues. These include (i) correlations among (near-)significant probes; (ii) similar probe sequences among (near-)significant probes; (iii) absence of regional effects at the target locus; (iv) association between the phenotype of interest and total signal intensities and (v) associations in type I OOB channels.

In the supplementary note we discuss these red flag signals in more detail, and show that varying sets of probes map to other disease-associated repeat sequences. We note that these red flags cannot conclusively confirm the presence or absence of cross-reactivity issues, however they do provide a strong incentive for cautious interpretation and follow-up of the results. Lastly, cross-reactivity is not the only phenomenon that can lead to spurious associations, most notably SNPs underlying the probe sequence also need to be taken into account (12,47). Ideally, given array artefacts as described here, researchers need to replicate their findings using a sequencing-based technique, even when array results appear consistent across batches. For example, bisulfite-pyrosequencing is a cost-effective method that has been successfully used to validate array-based findings (48,49).

To develop a full picture of the extent in which cross-hybridization can impact EWAS findings, additional studies will be needed. Due to the technical challenges involved in determining the exact *C9orf72* repeat length, we were unable to determine the relation between repeat length and amount of off-target signal (29,50). Future studies investigating this relation using long-read sequencing techniques for example would be valuable. In addition, the impact of factors such as GC-content and other experimental factors on cross-hybridization in DNA methylation arrays are worth further exploring. Moreover, it is interesting to note that the issues described here were limited to type I probes, especially given that previous studies have suggested that type II probes are more reliable than type I probes (51–53). However, notably fewer type II probes showed sequence overlap with the C9 repeat, and further studies would be required to establish whether type I and type II probes differ in terms of cross-hybridization.

## CONCLUSION

Illumina DNA methylation arrays provide a cost-effective approach to interrogate genome-wide DNA methylation levels in large samples and have proven to be a central tool in epigenetic research. However, results obtained using these arrays should be interpreted with caution as we empirically show that cross-hybridization can result in many false positive findings. Importantly, we found that excluding probes *a priori*, based on published annotations of cross-reactive probes, may fall short in preventing spurious associations. In this paper, we report several checks to identify said probes, and we expect that our approach will aid in preventing spurious associations in DNA methylation studies.

## DATA AVAILABILITY

Code is available at: https://github.com/pjhop/dnamarray_crossreactivity We implemented several functions that aid in detecting cross-reactive probes in an R package: https://github.com/pjhop/DNAmCrosshyb.

Individual-level DNA methylation data is available upon request in European Genome-phenome Archive (EGAS00001004587). All downstream data underlying tables and figures presented in the manuscript are available via https://doi.org/10.5281/zenodo.4110015.

## Supplementary Material

lqaa105_Supplemental_File

## Notes

**Disclaimer:** The views expressed are those of the authors and not necessarily those of the NHS, the NIHR or the Department of Health.
