# Supplementary material for: Cross-reactive probes on Illumina DNA methylation arrays: a large study on ALS shows that a cautionary approach is warranted in interpreting epigenome-wide association studies
Source: NAR Genom Bioinform. 2020 Dec 17;2(4):lqaa105. doi: 10.1093/nargab/lqaa105 (PMC7745769; doi:10.1093/nargab/lqaa105)
Supplement: lqaa105_Supplemental_File [file lqaa105_supplemental_file.pdf]

Contents

1 Supplementary Text **2**

2 Supplementary Tables **5**

3 Supplementary Figures **9**

4 Supplementary Note **20**

1 Supplementary Text

EWAS sensitivity analyses

We peformed several sensitivity analyses to assess how different analysis strategies affect the results:

1. Removing principal component (PC) outliers prior to the analysis. Samples with values >3 standard deviations from the mean of the first five principal components were removed.

2. Running a meta-analysis on the three experimental batches instead of a combined analysis. We ran the OSCA algorithm (as decribed above) on the three individual batches. We then performed an inverse-variance-weighted, fixed-effects meta-analysis using the *metagen()* function in the *meta* package [1].

3. Running a multivariate linear model adjusting for experimental batch, age, sex, smoking score, imputed cell fractions, the first 5 array-wide PCs and the first 5 control probe PCs.

4. Performing an EWAS on M-values (defined as $\log_2(\frac{\beta}{1-\beta})$) instead of β-values.

Changes in assumptions support the same conclusions

Here we present sensitivity analyses examining the impact of different assumptions on the results (see Figure S10 for an overview of the sensitivity analyses).

Unmethylated repeat sequence.

First, we assumed that the C9 repeat is fully methylated, and thus all C's in CpG-sites are retained. We reran the analyses assuming the repeat is fully unmethylated (all C's are converted to T's). We found that for several type I probes the unmethylated beads match a larger part of the unmethylated C9 repeat than the methylated beads match to the methylated C9 repeat (figure S11). Similarly, several type II probes match a larger part of the unmethylated C9 repeat than its methylated counterpart. In total, an additional 708 probes have a $\geq$14bp match to the C9 repeat when we assume the repeat is fully unmethylated. This can be explained by the lower sequence complexity of unmethylated DNA compared to methylated DNA: bisulfite conversion renders 'TG' and unmethylated 'CG' sequences indistinguishable from one another. None of the probes that had an increased match to the unmethylated C9 repeat were significant, presumably because the repeat is generally hypermethylated in C9 repeat carriers [2, 3].

Using Y bases.

Second, we assumed that the repeat is either fully methylated or fully unmethylated (i.e. the CpG in each repeat has the same methylation status). Alternatively, we can represent C's in CpG-sites by Y bases (Y = C or T in IUPAC code), thereby allowing any combination of methylation statuses within the body of the probe. For the methylated type I beads and type II probes this leads to an an increased match for some probes (figure S12-13). The C9 match length for unmethylated type I beads does not change when representing C's in CpG-sites by Ys (figure S12B). The probes with an increased C9 match upon using Y-bases were not significant, which may indicate that the C9 repeat is generally fully methylated.

Including regions flanking the C9 repeat.

Thirdly, we focused on the C9 repeat itself, and did not include the regions directly flanking the repeat. Probes may actually match both the repeat and the flanking region, leading to an increased hybridization length. We found that some probes indeed also partially match the regions flanking the C9 repeat, leading to an increased sequence match (Figure S14). However, none of these probes were significant. This presumably reflects the fact that the flanking sequences normally have a copy number of 2, whereas the repeat has many copies in carriers of the C9 repeat expansion (hundreds to thousands). Thus, in contrast to a match to the flanking sequences, a match to the repeat sequence can lead to many off-target hybridization events.

Allowing mismatches closer to the 3'end of the probe.

Finally, we performed inexact matching where we excluded matches with a mismatch/INDEL within 5 basepairs of the 3'end of the probe. We tested the validity of this cutoff by rerunning the analyses allowing mismatches/INDELs at any position in the probe. This led to 297 additional C9-mapping probes ($\geq$14bp match to either unmethylated or methylated C9 repeat, figure S15). One of these additional probes was significant in the OOB EWAS, implying that all significant OOB probes have a $\geq$14bp C9 match if we allow mismatches at any location in the probe. This indicates that off-target matches with a mismatch close to the 3'-end of the probe may still result in cross-hybridization.

C9-mapping probes show significant differences in signal intensity

Total signal intensity differences may be confounded by signal saturation effects, that is, for some probes β-values near 1 or 0 (i.e. fully methylated, or fully unmethylated) tend to have lower total signal intensities than β-values near 0.5 (intermediate methylation) [4]. This is because recorded intensities may truncate at high intensity levels, which is less likely to happen at intermediate methylation levels where the signal is roughly equally divided over two color channels. However, regardless of baseline β-values, we found similar total signal intensitiy differences across C9-mapping probes, indicating that there are true intensity differences between carriers and non-carriers (figure S16).

References

[1] Schwarzer, G. meta: An R package for meta-analysis. (2019).

[2] Xi, Z., Zhang, M., Bruni, A. C., Maletta, R. G., Colao, R., Fratta, P., Polke, J. M., Sweeney, M. G., Mudanohwo, E., Nacmias, B., and others (2015) The C9orf72 repeat expansion itself is methylated in ALS and FTLD patients. *Acta neuropathologica,* **129**(5), 715–727.

[3] Cohen-Hadad, Y., Altarescu, G., Eldar-Geva, T., Levi-Lahad, E., Zhang, M., Rogaeva, E., Gotkine, M., Bartok, O., Ashwal-Fluss, R., Kadener, S., Epsztejn-Litman, S., and Eiges, R. (November, 2016) Marked Differences in C9orf72 Methylation Status and Isoform Expression between C9/ALS Human Embryonic and Induced Pluripotent Stem Cells. *Stem Cell Reports,* **7**(5), 927–940.

[4] Zhou, W., Triche, T. J., Laird, P. W., and Shen, H. (July, 2018) SeSAMe: reducing artifactual detection of DNA methylation by Infinium BeadChips in genomic deletions. *Nucleic Acids Research,* **46**(20), e123.

[5] Zhou, W., Laird, P. W., and Shen, H. (2017) Comprehensive characterization, annotation and innovative use of Infinium DNA methylation BeadChip probes. *Nucleic Acids Research,* **45**(4), e22.

2 Supplementary Tables

Supplementary Table 1: Number of samples that fail on each QC measure in the 450k data. Note that samples may fail on multiple measures. The total represents the total number of samples that fail QC.

**Metric**	**Nr. Outliers**
MU	27
RG_ratio	0
GR_ratio	0
Sex Check	15
OP	20
Hyb	9
bscon	51
detectionP	36
beadNr	0
WGS - inbreeding	8
WGS - relatedness	12
IBS	38
Total	87

Supplementary Table 2: Number of samples that fail on each QC measure in the EPIC data. Note that samples may fail on multiple measures. The total represents the total number of samples that fail QC.

Metric	Nr. Outliers
MU	0
RG_ratio	0
GR_ratio	0
Sex Check	3
OP	0
Hyb	0
bscon	1
detectionP	0
beadNr	0
WGS - inbreeding	0
WGS - relatedness	0
IBS	0
Total	4

Supplementary Table 3: Baseline characteristics of the study population (450k, post-QC).

	ALS patients
Subjects, No.	1748
Male sex	1031 (59%)
C9orf72 carrier	119 (6.8%)

Supplementary Table 4: Baseline characteristics of the study population (EPIC, post-QC).

	ALS patients
Subjects, No.	437
Male sex	264 (60%)
C9orf72 carrier	33 (7.6%)

Supplementary Table 5: Probe sequences of the *trans* probes with sequence similarity to the bisulfite-converted C9 repeat.

Probe	Type	ProbeSeq (Methylated Bead is shown)
cg09994391	I	CTAAACCAAAACAAAAACTAAAACTAAAACCGAAACCGAAATCGAAACCG
cg19403339	I	TTAACCAAAACAATTCCCTCAACTCCGAATTAAAACCGAAACCGAAACCG
cg01370437	I	AATAAAAATAAAACCGTAATAAAACAAAAACCGTAACGAAACCGAAACCG
cg18002896	I	AAACTACAAAATCAAAACTACACCCAAAAAAAACCACGAAACCGAAACCG
cg15793563	I	ACCAAAACAAAAAACTAAACAAAACTACCCGAAAATCGAAACCGAAACCG
cg20307896	I	TCTCTCCATTCATTACTTTAACGAATATACAACAACCGAAATCGAAACCG
cg00521048	I	AATCGAATTATAAAACCCGAAAAACAAAATTTCGACCGAAACCGAAACCG
cg00801568	I	CCTCTAAAAAATTCAATAAAACGAAAATTATAAAACCGAAAACGAAACCG
cg16517021	I	CGAAATAAAAATTCCAATAACGACTACAAAAAACAACGAAACCGAAACCG
cg12078510	I	TAAAACCTTACCCTTTAAAACCGACAAAAAACGAAACCGAAACGAAACCG
cg22383472	I	TAAAAAATCGCGATACTAAATTCCACGAAAATAAAACCGAACCGAAACCG

Supplementary Table 6: Number of C9-mapping probes that would be excluded based on previous studies (Chen *et al.*, Naeem *et al.* and Zhou *et al.*)

Study	C9 mapping probes	Significant C9-mapping probes
Chen *et al.*	7/137	0/11
Naeem *et al.*	22/137	2/11
Zhou *et al.*	11/137	2/11
**Total**	**26/137**	**3/11**

Supplementary Table 7: Number of significant C9-mapping probes across different EWAS methods.

EWAS Method	# Significant	% Crossreactive
OSCA LOCO	18	61.1%
OSCA MOA	19	63.2%
OSCA MOMENT	12	41.7%
Linear Model*	19	63.2%
Linear Model* + Bacon	19	63.2%
SVA	21	66.7%
ISVA	21	66.7%

Supplementary Table 8: Number of 450k array probes that have a ≥14bp inexact match to different types of repeat sequences that have been associated with disease, and the number of these probes flagged as cross-reactive using a 30bp exact match as recommended by Zhou *et al.* [5]

Repeat Sequence	N crossreactive (>=14bp)*	N crossreactive (>=30bp)**
ATTCT	5	1
CAG	547	60
CTG	547	60
GAA	2	1
GCC	545	62
GCG	545	62
CGG	545	62

*Defined as a ≥14bp inexact match to the respective sequence (allowing 1 mismatch/INDEL >5bp from the 3'-end of the probe).

**Defined as a ≥30bp exact match to the respective sequence as recommended by Zhou *et al.* [5]

Supplementary Table 9: Number of EPIC array probes that have a ≥14bp inexact match to different types of repeat sequences that have been associated with disease, and the number of these probes flagged as cross-reactive using a 30bp exact match as recommended by Zhou *et al.*

Repeat Sequence	N crossreactive (>=14bp)*	N crossreactive (>=30bp)**
ATTCT	12	2
CAG	710	70
CTG	710	70
GAA	5	1
GCC	696	71
GCG	696	71
CGG	696	71

*Defined as a ≥14bp inexact match to the respective sequence (allowing 1 mismatch/INDEL >5bp from the 3'-end of the probe).

**Defined as a ≥30bp exact match to the respective sequence as recommended by Zhou *et al.* [5]

3 Supplementary Figures

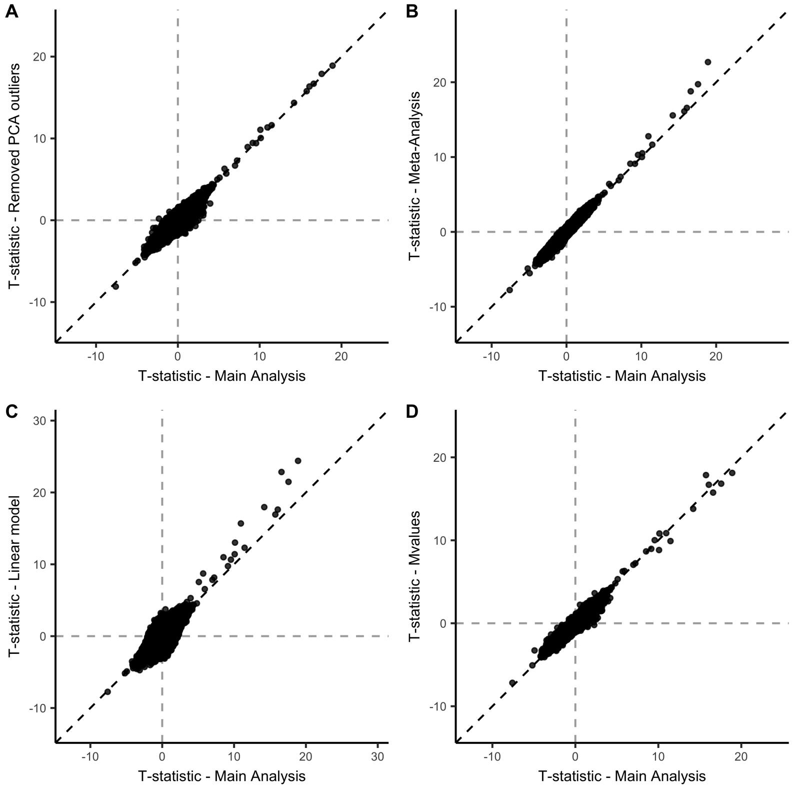

Supplementary Figure 1: EWAS sensitivity analyses, described in the supplementary text. **(A)** Test-statistics after removing 47 PCA-outliers (y-axis) compared with the test-statistics from the main analysis (x-axis). MLMA LOCO was used for both EWASs. **(B)** Test-statistics from the meta-analysis on the three experimental batches in the data (y-axis) compared with the test-statistics from the main analysis (x-axis). MLMA LOCO was used for both the main analysis and the EWASs in the individual experimental batches. **(C)** Test-statistics from a multivariate linear model (y-axis) compared with the test-statistics from the main analysis (x-axis, MLMA LOCO). **(D)** Test-statistics from a MLMA LOCO results using M-values (y-axis) instead of β-values (x-axis).

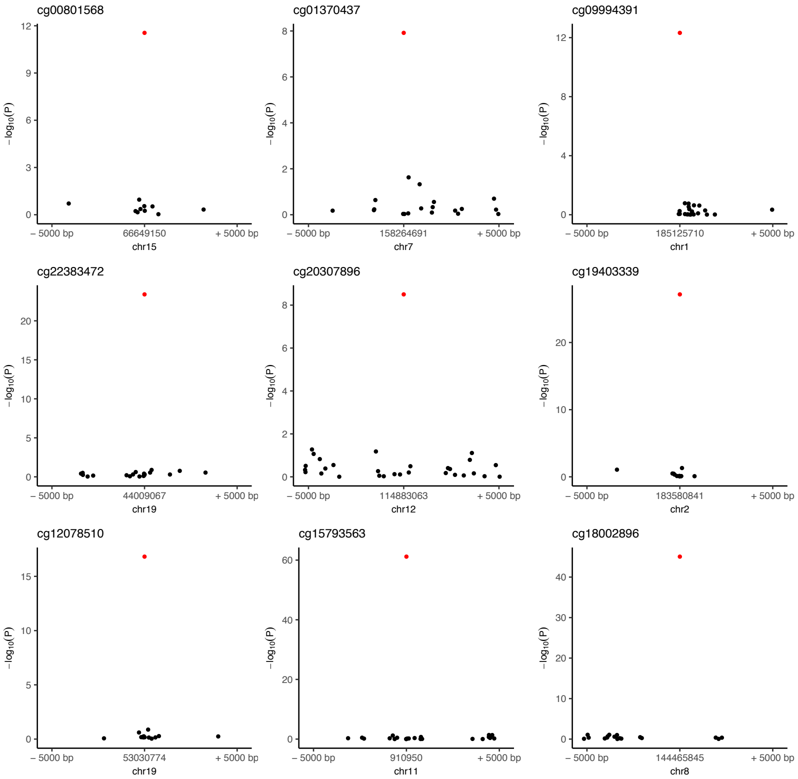

Supplementary Figure 2: Locus plots of nine *trans* CpGs associated with *C9orf72* status.

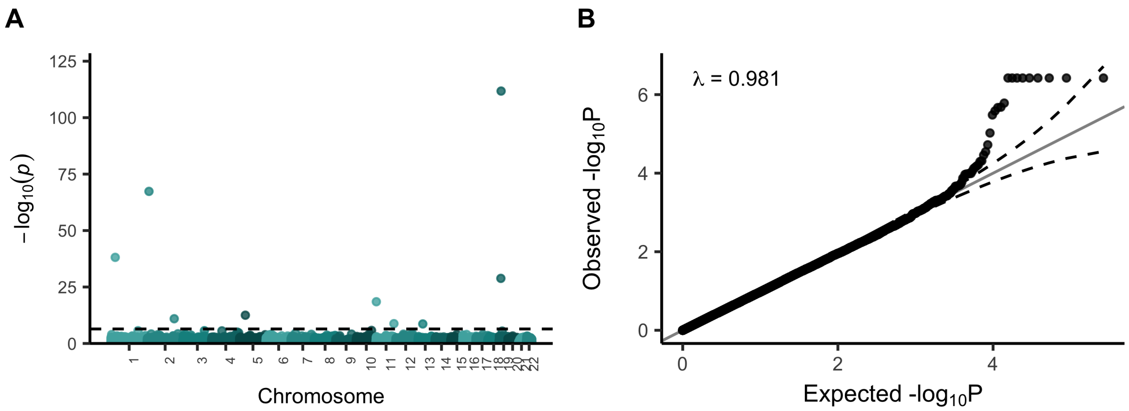

Supplementary Figure 3: Out-of-band (OOB) EWAS. Epigenome-wide association study (EWAS) on *C9orf72* status within ALS patients where β-values were calculated using the (OOB) type I probe intensities. **(A)** Manhattan plot comparing association P-values ($-\log_{10}(P)$, y-axis) and genomic location (x-axis). The dashed line indicates the genome-wide significance threshold. **(B)** QQ-plot showing observed P-values ($-\log_{10}(P)$, y-axis) against the expected distribution under the null (x-axis). For presentation purposes P-values $<3.8 \times 10^{-7}$ are plotted as 3.8×10^{-7}.

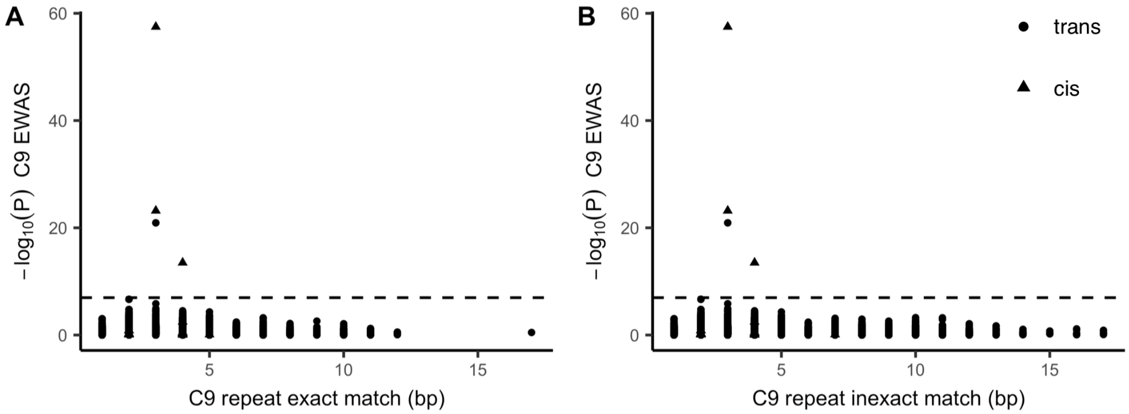

Supplementary Figure 4: Type II probes. Comparison between P-values from the *C9orf72* EWAS and match (bp) between the 3'-subsequence and the bisulfite-converted *C9orf72* hexanucleotide repeat expansion. We assumed that the repeat was completely methylated. **(A)** Association P-values($-\log_{10}(P)$, y-axis) vs. 3'subsequence match length (x-axis). **(B)** Association P-values ($-\log_{10}(P)$, y-axis) vs. 3'subsequence match length (x-axis), where we allowed one mismatch/INDEL (>5bp from the 3' end of the probe).

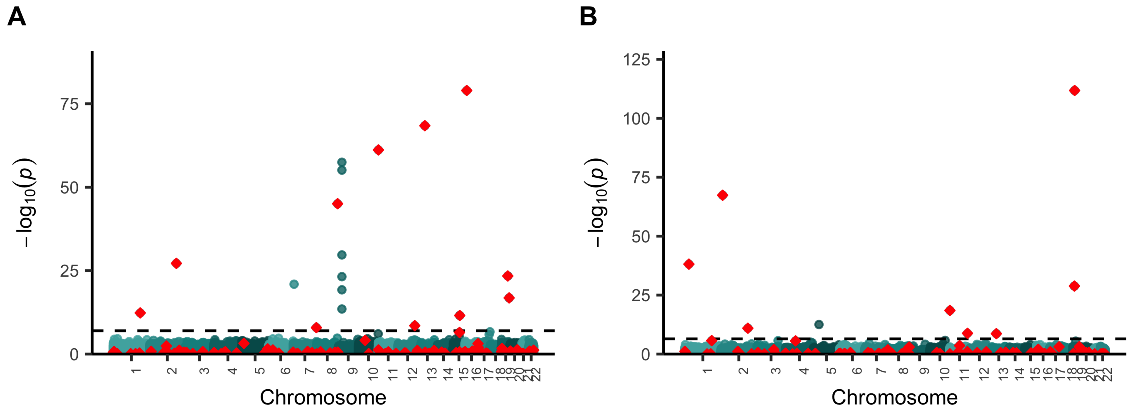

Supplementary Figure 5: Epigenome-wide association study on *C9orf72* status within ALS patients, where probes (N = 137) with ≥14bp 3'-subsequence match to the bisulfite converted *C9orf72* repeat are highlighted. **(A)** Manhattan plot comparing association P-values ($-\log_{10}(P)$, y-axis) and genomic location (x-axis). The dashed line indicates the genome-wide significance threshold (1.1×10^{-7}) **(B)** Manhattan plot comparing association P-values ($-\log_{10}(P)$, y-axis) and genomic location (x-axis) from an EWAS on out-of-band (OOB) β-values. The dashed line indicates the genome-wide significance threshold (3.8×10^{-7}).

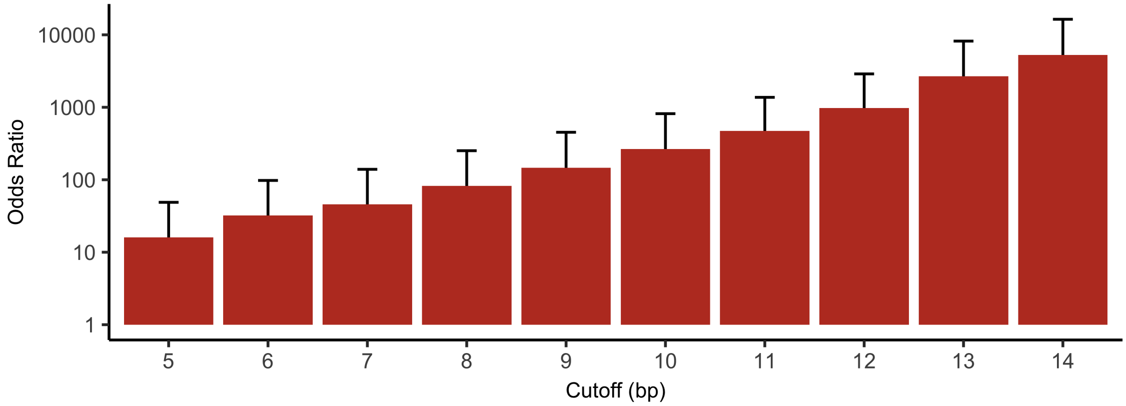

Supplementary Figure 6: Enrichment for probes that partially match the C9 repeat among loci that were significant in the C9 EWAS. Enrichments were calculated for different cutoffs to define probes that partially match the C9 repeat. We performed Fisher's exact test to obtain confidence intervals (95%).

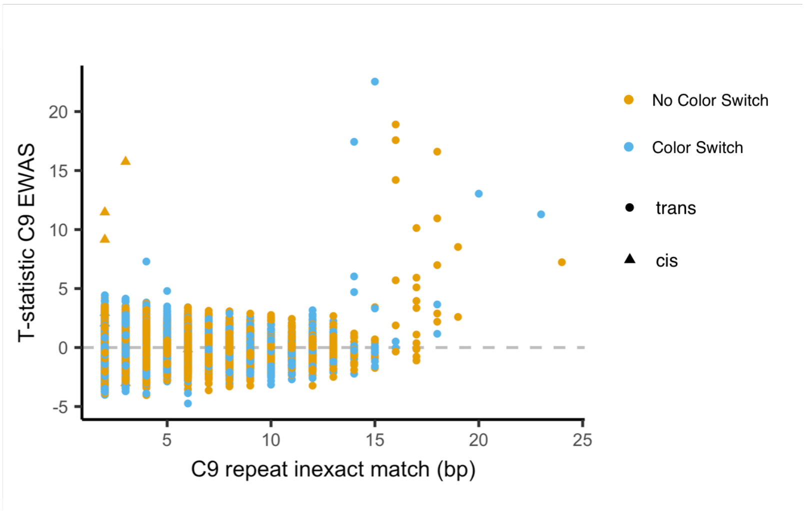

Supplementary Figure 7: Comparison between association test-statistics from the *C9orf72* EWAS (y-axis) and inexact match (bp) between the 3'-subsequence and the bisulfite-converted *C9orf72* hexanucleotide repeat expansion. Test-statistics from the EWAS on the out-of-band (OOB) β-values were used in case of a predicted color-channel switch.

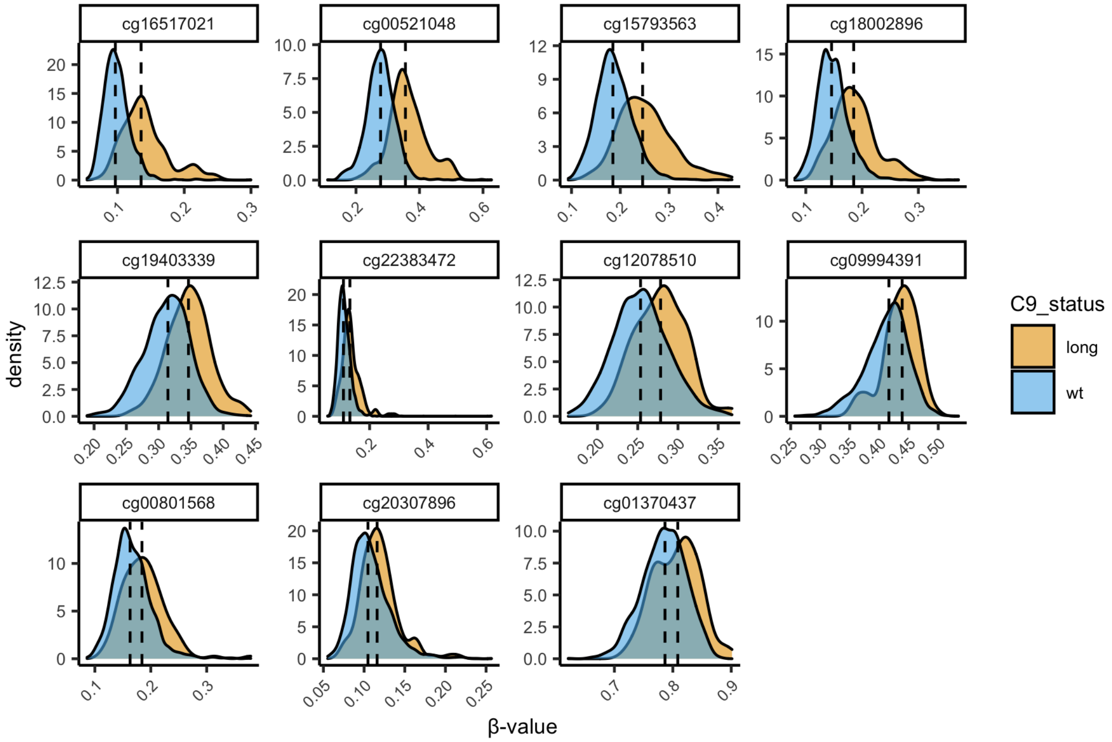

Supplementary Figure 8: Density plots of the in-band β-values of the significant C9-mapping probes. The dashed lines indicate the median β-value for the non-carriers and carriers of the C9 repeat expansion respectively.

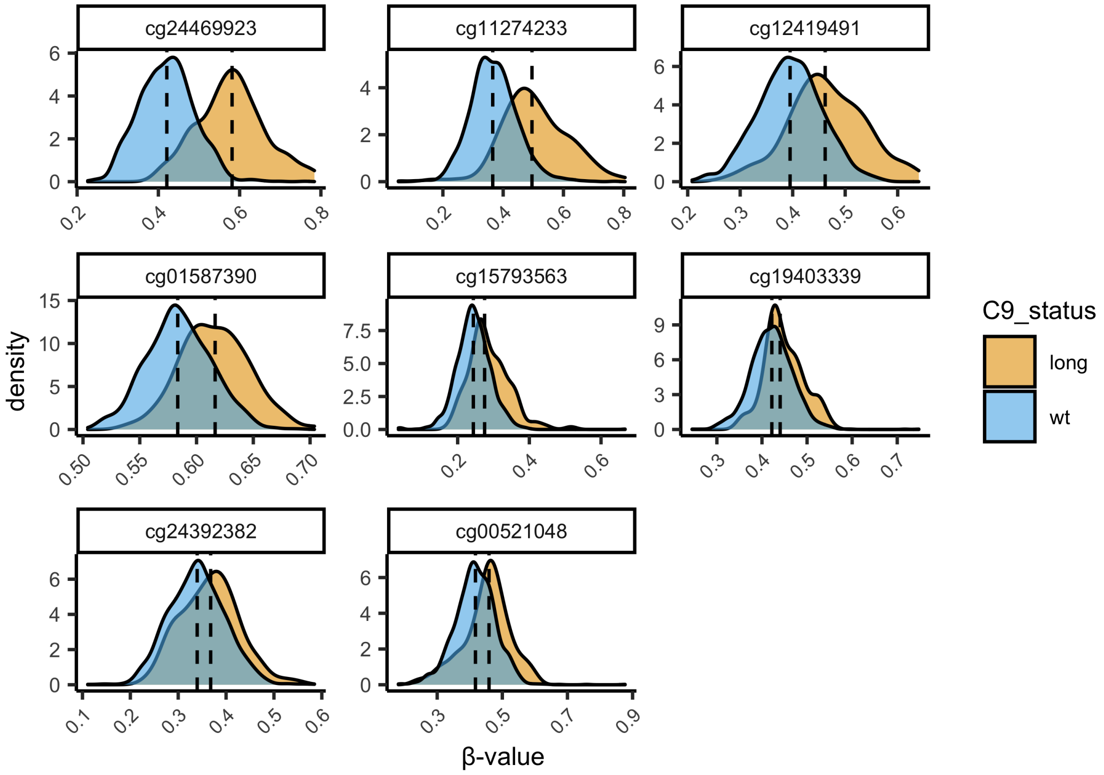

Supplementary Figure 9: Density plots of the out-of-band β-values of the significant C9-mapping probes. The dashed lines indicate the median β-value for the non-carriers and carriers of the C9 repeat expansion respectively.

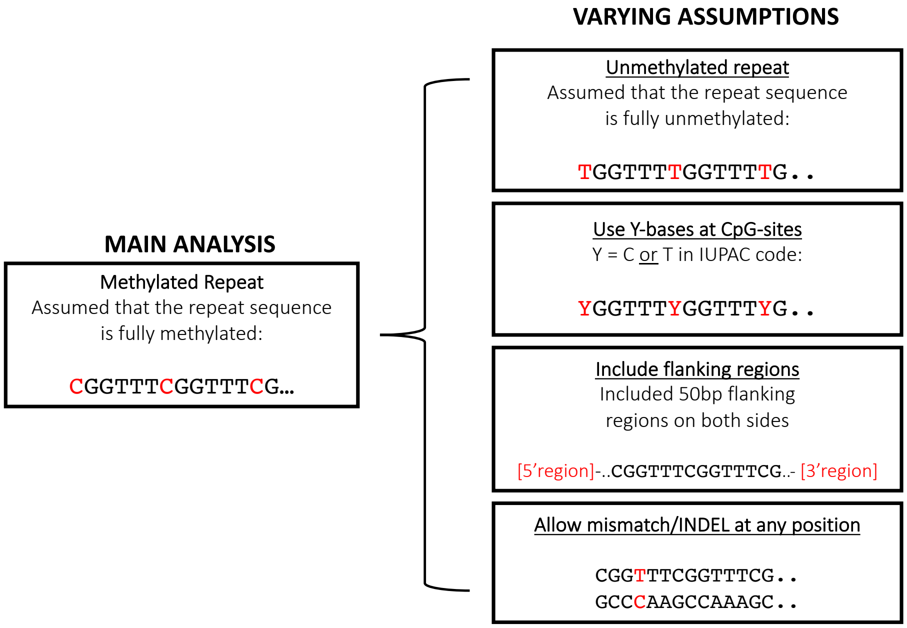

Supplementary Figure 10: Overview of the different assumptions tested in the paragraph 'Changes in assumptions support the same conclusions' which is included as supplementary text.

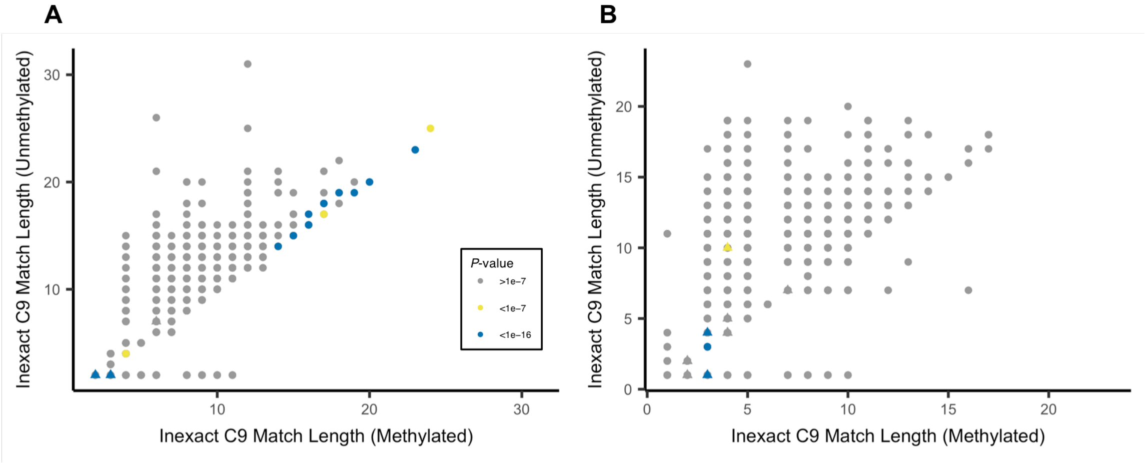

Supplementary Figure 11: Comparison between match (bp) between the probe sequence and the methylated C9 repeat and the unmethylated C9 repeat respectively. **(A)** Type I probes. Comparison between the 3'-subsequence match of unmethylated type I beads to the unmethylated C9 repeat (y-axis) and the 3'-subsequence match of methylated type I beads to the methylated C9 repeat (x-axis). Points are colored by significance levels in the C9 EWAS. **(B)** Type II probes. Comparison of the 3'-subsequence match to the unmethylated C9 repeat (y-axis) and the 3'-subsequence match to the methylated C9 repeat (x-axis).

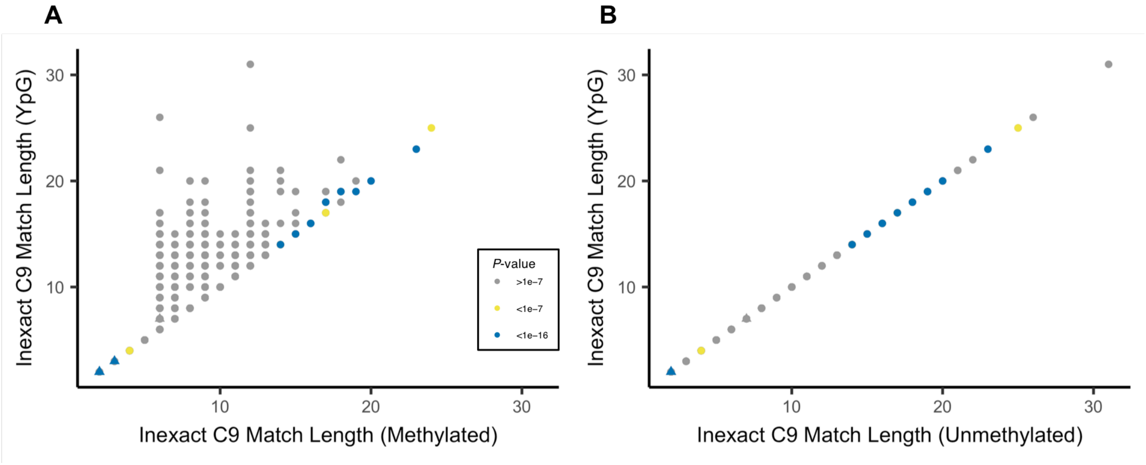

Supplementary Figure 12: Increase in match length for type I probes when Cs in CpG-sites are represented by Y bases (Y = C or T in IUPAC code), instead of assuming that the C9 repeat is either fully methylated or fully unmethylated. **(A)** Comparison between the 3'-subsequence match of methylated type I beads to the C9 repeat where C's in CpG-sites are represented by Y bases (y-axis) and 3'-subsequence match of methylated type I beads to the fully methylated C9 repeat (x-axis). **(B)** Comparison between the 3'-subsequence match of unmethylated type I beads to the C9 repeat where C's in CpG-sites are represented by Y bases (y-axis) and 3'-subsequence match of unmethylated type I beads to the fully unmethylated C9 repeat (x-axis).

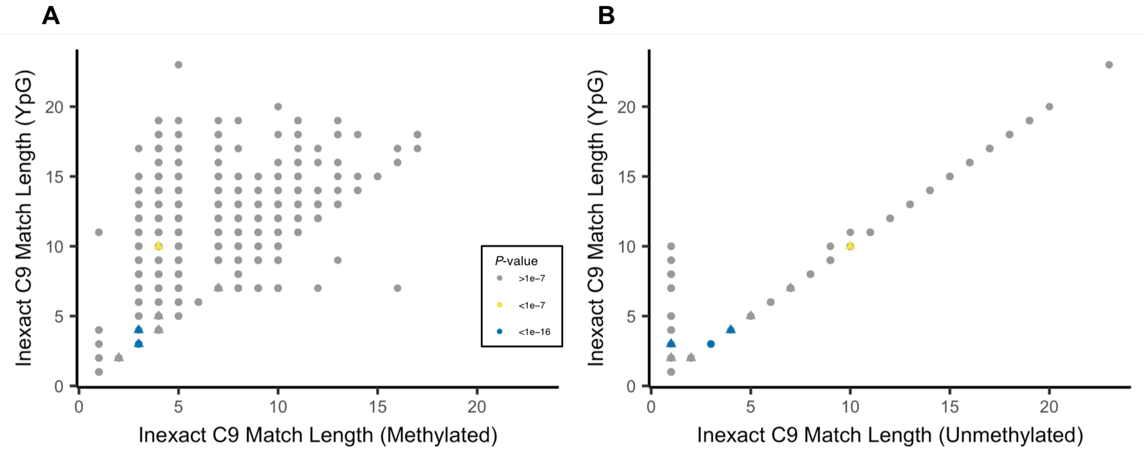

Supplementary Figure 13: Increase in match length for type II probes when Cs in CpG-sites are represented by Y bases (Y = C or T in IUPAC code), instead of assuming that the C9 repeat is either fully methylated or unmethylated **(A)** Comparison between the 3'-subsequence match of type II probes to the C9 repeat where C's in CpG-sites are represented by Y bases (y-axis) and 3'-subsequence match of type II probes to the fully methylated C9 repeat (x-axis). **(B)** Comparison between the 3'-subsequence match of type II probes to the C9 repeat where C's in CpG-sites are represented by Y bases (y-axis) and 3'-subsequence match of type II probes to the fully unmethylated C9 repeat (x-axis).

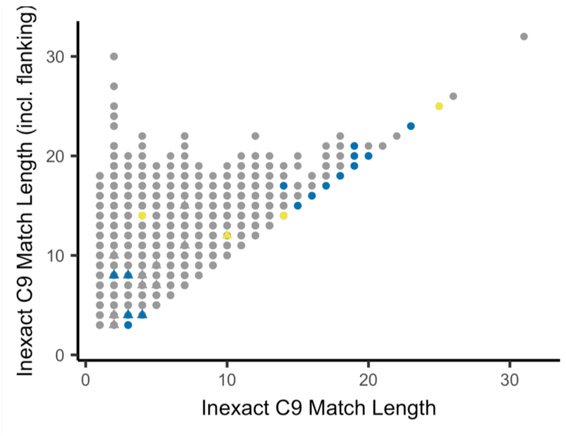

Supplementary Figure 14: Increase in match length for type I (methylated and unmethylated beads) and type II probes when the regions flanking the C9 repeat are included in addition to the C9 repeat itself.

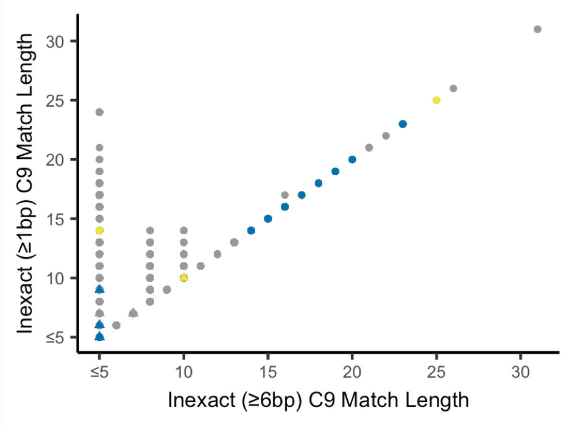

Supplementary Figure 15: Increase in match length for type I (methylated and unmethylated beads) and type II probes when we allow a mismatch/INDEL at any position in the probe instead of allowing mismatches/INDELs $\geq$6bp from the 3'-end of the probe.

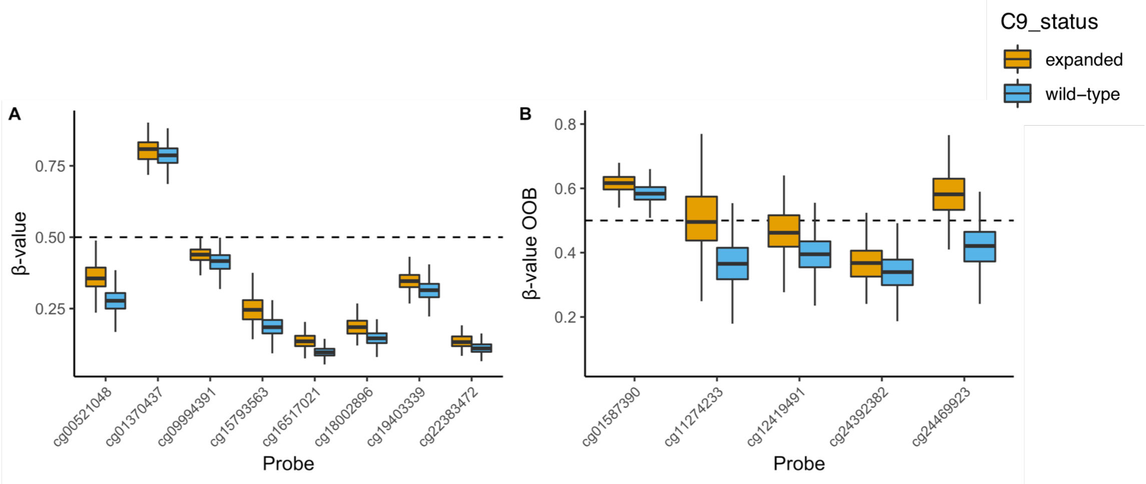

Supplementary Figure 16: Boxplots of β-values of probes for which total signal intensity (M + U) was associated with C9 status. Measurements with β-values closer to 0.5 may show higher total signal intensities because they are less affected by signal saturation. **(A)** In-band β-values. Although for most probes the β-values of carriers of the C9 expansion have β-values closer to 0.5, for one probe (cg1370437) the β-values of non-carriers (wild-type) are closer to 0.5. **(B)** Out-of-band (OOB) β-values.

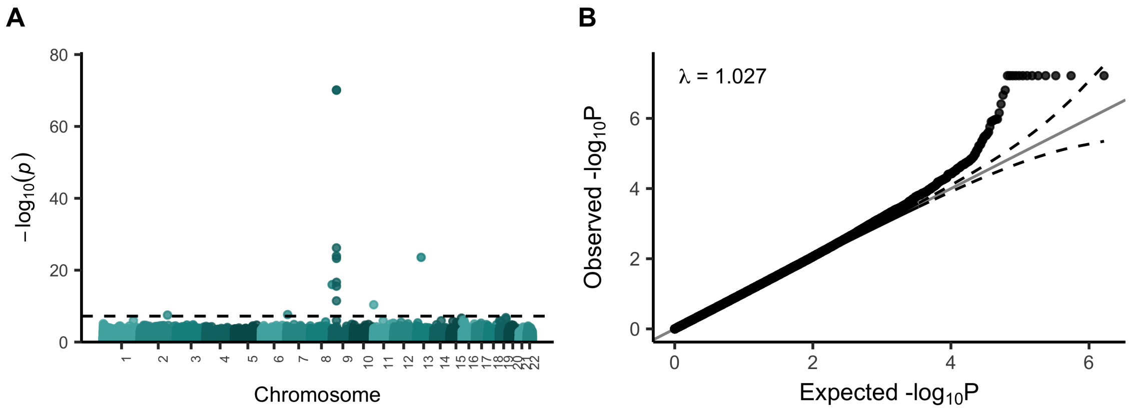

Supplementary Figure 17: EWAS in EPIC cohort on *C9orf72* status within ALS patients. **(A)** Manhattan plot comparing association P-values ($-\log_{10}(P)$, y-axis) and genomic location (x-axis). The dashed line indicates the genome-wide significance threshold (6.1×10^{-8}). **(B)** QQ-plot showing observed P-values ($-\log_{10}(P)$, y-axis) against the expected distribution under the null (x-axis). For presentation purposes P-values $<6.1 \times 10^{-8}$ are plotted as 6.1×10^{-8}.

Supplementary Note

Paul Hop

10/2020

Contents

- Data availability 1
  - Introduction 1
  - 1. Off-target sequence matches 1
  - 2. Correlations among (near-)significant probes 5
  - 3. Sequence overlap between (near-)significant probes 6
  - 4. Absence of regional effects at the target locus 8
  - 5. Associations in type I OOB channels 10
  - 6. Associations between total intensity and the phenotype 11
  - 7. Other issues 11
  - References 11

Supplementary note corresponding to the manuscript: "Crossreactive probes on Illumina DNA methylation arrays: a large study on ALS shows that a cautionary approach is warranted in interpreting epigenome-wide association studies".

Data availability

The data used in this markdown can be downloaded from: https://doi.org/10.5281/zenodo.4110015. This script can be reproduced using the deposited data as follows:
- Clone this repository: < git clone https://github.com/pjhop/dnamarray_crossreactivity.git >
- Download the data ('data.zip') and place it in the 'dnamarray_crossreactivity' folder.
- Unzip the data.zip folder

Introduction

In the manuscript, we discuss several analytical checks that can aid in identifying cross-reactive probes. Here, we provide additional comments on these checks, discussing what they can and cannot tell about potential cross-reactivity issues. Moreover, we provide example R code for each check. For all scripts used in the manuscript, please visit: http://github.com/pjhop/dnamarray_crossreactivity. We wrapped several scripts into an R package which we also use in this document. The package is available at: http://github.com/pjhop/DNAmCrosshyb.

1. Off-target sequence matches

As suggested by previous studies, we recommend checking significant probes for off-target matches. The annotation files published by Zhou *et al.* (Zhou, Laird, and Shen 2017) provide a good starting point and can be downloaded here: https://zwdzwd.github.io/InfiniumAnnotation These files contain information on the number of off-target matches at different match lengths for each probe, and other useful information such as SNP-masking and probe-mapping quality (also see 7. Other issues).
The authors recommend removing probes with one or more >=30bp off-target matches. However, we note that:

1. Sequence matches <30bp can be sufficient for detectable cross-hybridization. This is especially the case when those off-target matches measure similar DNA methylation levels, for example when the off-target matches are located in a repeat region. It is not straightforward to determine a exact cutoff. For example, many 20-25bp off-target matches may be more problematic (for example when a probes maps to a tandem repeat) than 1 30bp off-target match. We therefore recommend careful examination of the results, the other checks described in this document can help in identifying issues.
2. Imperfect matches off-target matches (i.e. allow mismatched INDELs) may lead to spurious associations. Additionally, by allowing a mismatch you also account for probes that may match an off-target region only when a SNP is present.
3. Genetic variation (especially structural variation such as tandem repeats) associated with the phenotype should be taken into account, since these are not taken into account by mapping probes to the reference genome.

Examples Zhou annotations:

```
# The MASK_sub30_copy column indicates that the probe has one or more >=30 bp off-target matches.
# The MASK_mapping column indicates that the probe has a low quality score
zhou_450k %>% dplyr::select(probeID, MASK_sub30_copy, MASK_mapping) %>%
  dplyr::filter(MASK_sub30_copy | MASK_mapping) %>%
  head()
```

```
## # A tibble: 6 x 3
##   probeID    MASK_sub30_copy MASK_mapping
##   <chr>      <lgl>           <lgl>
## 1 cg13869341 TRUE            TRUE
## 2 cg14008030 FALSE           TRUE
## 3 cg12045430 TRUE            TRUE
## 4 cg20826792 FALSE           TRUE
## 5 cg00381604 FALSE           TRUE
## 6 cg20253340 TRUE            TRUE
```

Mapping probes to a non-reference sequence using the `DNAmCrosshyb` package (GGCCC hexanucleotide repeat)

```
# Map probes to the C9orf72 hexanucleotide repeat
repeat_sequence <- paste(rep("GGCCCC", 10), collapse="")

matches_c9 <- map_probes_sequence(sequence = repeat_sequence, next_base = "G", prev_base = "C",
                            array = "450k", min_width = 14,
                            max_width = 30, allow_indel = FALSE,
                            allow_mismatch = FALSE, step_size = 1,
                            use_Y = FALSE, methylation_status = "methylated")

data.frame(matches_c9 %>% dplyr::arrange(desc(width))) %>% head()
```

```
##        Probe start end  strand width sbe_site mismatch_pos max_mismatch_pos
## 1 cg01587390     6  25 forward    20        T           NA               NA
## 2 cg01587390    12  31 forward    20        T           NA               NA
## 3 cg01587390    18  37 forward    20        T           NA               NA
## 4 cg01587390    24  43 forward    20        T           NA               NA
## 5 cg01587390    30  49 forward    20        T           NA               NA
## 6 cg01587390    36  55 forward    20        T           NA               NA
##   n_mismatch indel_pos width_incl_indel          sequence_bs        Type2
## 1          0        NA               NA CGGTTTCGGTTTCGGTTTCG I_Methylated
## 2          0        NA               NA CGGTTTCGGTTTCGGTTTCG I_Methylated
```

```
## 3          0        NA                NA CGGTTTCGGTTTCGGTTTCG I_Methylated
## 4          0        NA                NA CGGTTTCGGTTTCGGTTTCG I_Methylated
## 5          0        NA                NA CGGTTTCGGTTTCGGTTTCG I_Methylated
## 6          0        NA                NA CGGTTTCGGTTTCGGTTTCG I_Methylated
##   channel
## 1     OOB
## 2     OOB
## 3     OOB
## 4     OOB
## 5     OOB
## 6     OOB
```

Mapping probes to several disease-associated repeat-sequences:

```
## run for several sequences, takes 30-40 minutes to run
sequences <- list(
  ATTCT = list(
    sequence = paste(rep("ATTCT", 15), collapse=""),
    prev_base = "T",
    next_base = "A"
  ),
  CAG = list(
    sequence = paste(rep("CAG", 20), collapse=""),
    prev_base = "G",
    next_base = "C"
  ),
  CTG = list(
    sequence = paste(rep("CTG", 20), collapse=""),
    prev_base = "G",
    next_base = "C"
  ),
  GAA = list(
    sequence = paste(rep("GAA", 20), collapse=""),
    prev_base = "A",
    next_base = "G"
  ),
  GCC = list(
    sequence = paste(rep("GCC", 20), collapse=""),
    prev_base = "C",
    next_base = "G"
  ),
  GCG = list(
    sequence = paste(rep("GCG", 20), collapse=""),
    next_base = "G",
    prev_base = "G"
  ),
  CGG = list(
    sequence = paste(rep("CGG", 20), collapse=""),
    next_base = "C",
    prev_base = "G"
)
)

run <- function(lst) {
  matches <- map_probes_sequence(sequence = lst[["sequence"]],
```

```
                                  next_base = lst[["next_base"]],
                                  prev_base = lst[["prev_base"]],
                       array = "450k", min_width = 14, max_width = 20,
                       allow_indel = TRUE,
                       allow_mismatch = TRUE, step_size = 1,
                       use_Y = TRUE)
  matches
}

matches_all <- purrr::map(sequences, .f = run)
names(matches_all) <- names(sequences)

# Check number of probes with >=14bp match for each sequence
# Check if filtered by Zhou et al.
zhou <- zhou_450k %>% dplyr::filter(MASK_sub30_copy | MASK_mapping)
make_overview <- function(sequence, matches_all) {
  check <- matches_all[[sequence]] %>% dplyr::filter(width >= 14)
  tibble(sequence = sequence, n_cr = dplyr::n_distinct(check$Probe),
         n_cr_zhou = sum(unique(check$Probe) %in% zhou$probeID))
}
overview <- purrr::map_df(names(matches_all), .f = make_overview, matches_all=matches_all)
overview
```

```
## # A tibble: 7 x 3
##   sequence  n_cr n_cr_zhou
##   <chr>    <int>     <int>
## 1 ATTCT        5         1
## 2 CAG        547        60
## 3 CTG        547        60
## 4 GAA          2         1
## 5 GCC        545        62
## 6 GCG        545        62
## 7 CGG        545        62
```

Mapping probes to the reference genome sequence, allowing mismatches and identifying off-target matches as small as 15bp. Bisulfite-converted reference genomes can be generated using the following scripts: https://github.com/pjhop/DNAmCrosshyb/blob/master/data-raw/bisulfite_convert_hg19.R and https://github.com/pjhop/DNAmCrosshyb/blob/master/data-raw/bisulfite_convert_hg38.R

Bisulfite-converted genomes in the R .rds file format are available at: https://doi.org/10.5281/zenodo.4088019

```
# takes 30-40 minutes to run
probes <- c("cg00005164", "cg12344104", "cg25521682",
            "cg27660038", "cg20554142", "cg03890998",
            "cg00947801")
matches <- map_probes(probes,
                      path = "../data/genome_bs/hg19",
                      chromosomes = "all",
                      min_width = 15,
                      max_width = 20,
                      step_size = 5,
                      allow_mismatch = TRUE,
                      allow_INDEL = FALSE,
                      cores = 1
                     )
```

```r
head(matches %>% dplyr::arrange(desc(width)) %>% data.frame())
```

```
##         Probe chr     start       end  strand next_base mismatch_pos Type2 width
## 1 cg12344104   1  62850945  62850964 forward         T            8    II    20
## 2 cg12344104   1  90067416  90067435 forward         A           20    II    20
## 3 cg12344104   1 154089442 154089461 forward         A            8    II    20
## 4 cg12344104   1 178803544 178803563 forward         T            2    II    20
## 5 cg25521682   1  40925714  40925733 forward         T            1    II    20
## 6 cg25521682   1  61961145  61961164 forward         G            5    II    20
##   channel
## 1    <NA>
## 2    <NA>
## 3    <NA>
## 4    <NA>
## 5    <NA>
## 6    <NA>
```

Number of matches per probe:

```r
nr_matches <- get_nr_matches_per_probe(matches)
nr_matches
```

```
## # A tibble: 7 x 3
## # Groups:   Probe [7]
##   Probe        bp15   bp20
##   <chr>       <int>  <int>
## 1 cg00005164 179986 145507
## 2 cg00947801  98421   9790
## 3 cg03890998  79769   7686
## 4 cg12344104  60427    140
## 5 cg20554142 282145   9069
## 6 cg25521682   5810    138
## 7 cg27660038   4342     17
```

Overlap between off-target matches and repeat sequences:

```r
matches <- find_repeat_overlaps(matches, genome_build = "hg19", min_overlap = "any")
head(matches %>% data.frame())
```

```
##         Probe chr  start    end  strand next_base mismatch_pos Type2 width
## 1 cg12344104   1  18881  18895 forward         Y            7    II    15
## 2 cg12344104   1  78306  78320 forward         T            8    II    15
## 3 cg12344104   1 333815 333829 forward         T            2    II    15
## 4 cg12344104   1 357053 357067 forward         A            5    II    15
## 5 cg12344104   1 457041 457055 forward         T            9    II    15
## 6 cg12344104   1 554449 554463 forward         T           10    II    15
##   channel repeat_overlap
## 1    <NA>          FALSE
## 2    <NA>           TRUE
## 3    <NA>           TRUE
## 4    <NA>           TRUE
## 5    <NA>           TRUE
## 6    <NA>          FALSE
```

2. Correlations among (near-)significant probes

Correlations among distal (near-)significant probes may be indicative of a technical issues.

Notes

1. Correlations may also be caused by biological factors. For example, a mutation in a gene that regulates DNA methylation, such as *DNMT3A*, may result in correlated differentially methylated CpG-sites. Moreover, confounding factors, such as batch effects and white blood cell composition, often result in groups of correlated probes. Although in the latter case correlations are not caused by cross-hybridization, they do often represent unwanted variation. In case of correlated probes, checking for sequence overlap will help in distinguishing between cross-hybridization-related correlation and other sources. The `get_probe_overlaps()` function in the `DNAmCrosshyb` package can be used to do this (see example in next section).

Example Example of a correlation heatmap of the sites significantly associated with C9 status in our study. Note that 2 blocks of probes are correlated. The left upper corner includes the *trans* probes that we found to be cross-reactive (to the C9 repeat). The middle block consists of four probes that are all located within the same region (CpG island within the *C9orf72* gene).

```
# Heatmap
pal <- viridis::viridis(n = 200)
breaksList = seq(-1,1, by = 0.01)
heatmap <- pheatmap(cor(t(beta_sig), use = "complete.obs"),
                            color = pal,
                            breaks = breaksList,
                            width = 3, height = 4,
                            fontsize = 5)
```

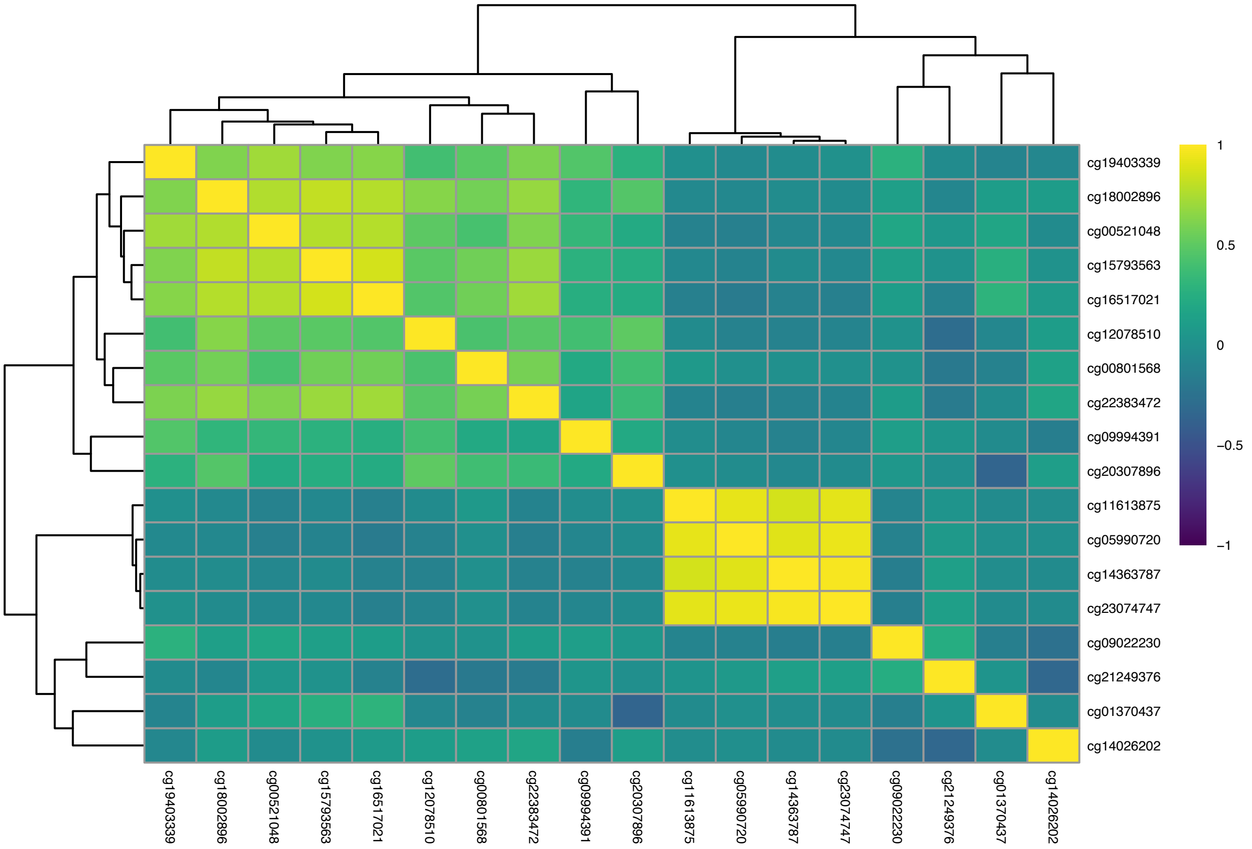

3. Sequence overlap between (near-)significant probes

This point is related to the previous one: it is suspicious when significant sites share similar probe sequences.

Examples The `get_probe_overlaps` function in the `DNAmCrosshyb` package can be used to check for overlapping 3'-subsequences in a set of probes. Here we apply this function to the probes that were significant in the C9 EWAS. We found that all *trans* probes that were correlated had a >=10bp overlap with each other.

```
overlaps <- get_probe_overlaps(c(mlma_sig$Probe, mlma_oob_sig$Probe))
```

```
## Calculating overlaps..
```

```
## Done!
```

```
overlaps %>% dplyr::arrange(desc(overlaps$overlap))
```

```
## # A tibble: 1,892 x 6
##    Probe_Index Bead_Index      Probe_Target Bead_Target     overlap mismatch_pos
##    <chr>       <chr>           <chr>        <chr>             <int>        <int>
##  1 cg18002896  cg18002896_unm~ cg24392382   cg24392382_unm~      25           13
##  2 cg24392382  cg24392382_unm~ cg18002896   cg18002896_unm~      25           13
##  3 cg18002896  cg18002896_unm~ cg16517021   cg16517021_unm~      24           16
##  4 cg16517021  cg16517021_unm~ cg18002896   cg18002896_unm~      24           16
##  5 cg18002896  cg18002896_met~ cg16517021   cg16517021_met~      24           16
##  6 cg16517021  cg16517021_met~ cg18002896   cg18002896_met~      24           16
##  7 cg09994391  cg09994391_unm~ cg01587390   cg01587390_unm~      21            9
##  8 cg12419491  cg12419491_unm~ cg01587390   cg01587390_unm~      21            7
##  9 cg19403339  cg19403339_unm~ cg00801568   cg00801568_unm~      21            9
## 10 cg19403339  cg19403339_unm~ cg01587390   cg01587390_unm~      21           20
## # ... with 1,882 more rows
```

Plot overlaps:

```
ggplot(overlaps %>% filter(Probe_Index != Probe_Target),
       aes(x = overlap)) +
  geom_histogram() +
  theme_classic() +
  xlab("Pair-wise probe overlap (bp)") +
  geom_vline(xintercept = 14, linetype = "dashed", color = "red") +
  theme(text = element_text(size=13))
```

```
## `stat_bin()` using `bins = 30`. Pick better value with `binwidth`.
```

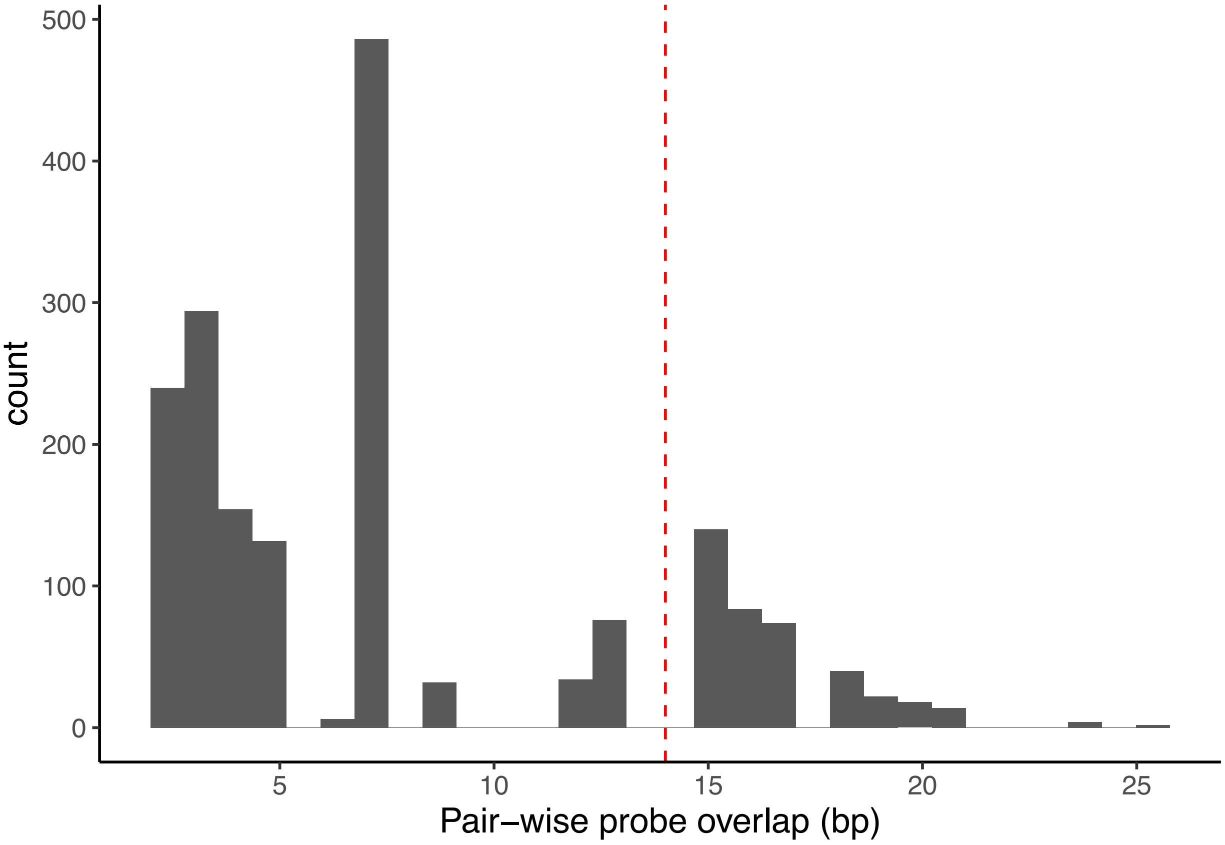

Check for each probe with how many other probes is has a >=10bp sequence overlap.

```
check <- overlaps %>% dplyr::group_by(Bead_Index) %>% summarize(n = sum(overlap >= 10))
```

```
## `summarise()` ungrouping output (override with `.groups` argument)
```

```
check %>% dplyr::filter(n > 1)
```

```
## # A tibble: 34 x 2
##    Bead_Index                   n
##    <chr>                    <int>
##  1 cg00521048_methylated       16
##  2 cg00521048_unmethylated     16
##  3 cg00801568_methylated       14
##  4 cg00801568_unmethylated     14
##  5 cg01370437_methylated       16
##  6 cg01370437_unmethylated     16
##  7 cg01587390_methylated       16
##  8 cg01587390_unmethylated     16
##  9 cg09994391_methylated       14
## 10 cg09994391_unmethylated     14
## # ... with 24 more rows
```

4. Absence of regional effects at the target locus

We often expect that DNA methylation changes are not restricted to specific CpG-sites, but extend to surrounding regions. Therefore, a 'lonely hit', i.e. a signification association without any signal in the surrounding CpGs may indicate a technical issue. Instead of focusing on differentially methylated positions (DMPs), differentially methylated regions (DMRs) may be more biologically relevant and more robust to

technical issues (Mill and Heijmans 2013)

Notes

1. Illumina DNA methylation arrays have a limited resolution, CpG-sites surrounding a target locus may not be measured. Therefore, identifying DMRs cannot completely replace DMPs, since a DMP may be reflective of a regional effect that is not measured and in certain cases DNA methylation changes may be restricted to only one or a feq sites.

Examples The `locusplot()` function from the `DNAmCrossHyb` package can be used to plot a significant CpG-site and its neighbouring CpG-sites.

(Likely) True positive First, we show a locus plot of the C9 island, which is very likely to be a true positive (found in different studies/tissues/techniques). Here, there is a regional effect, strengthening the evidence for a real biological effect:

```
# First an example of probes in the C9 island,
# these probes are very likely to be true positives (found in different # studies/tissues/techniques)
locusplot("cg05990720", stats = mlma,
         windowsize = 5000, significance_line = TRUE)
```

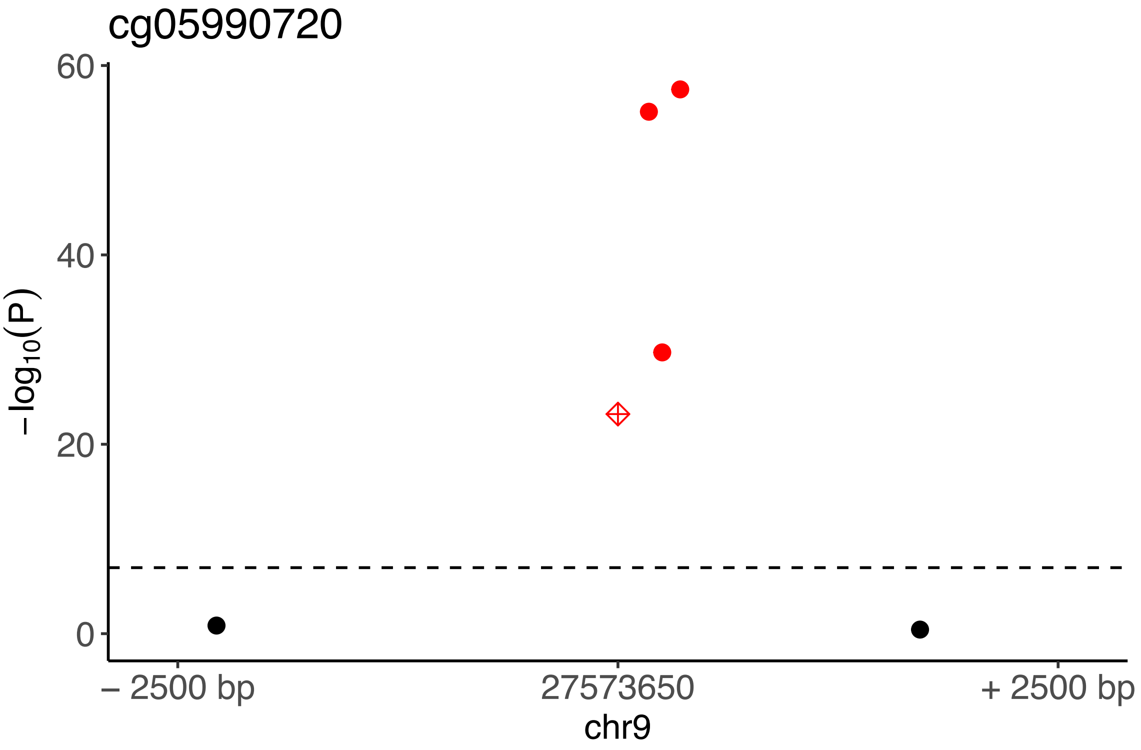

False positive Here, we plot one of the significant *trans* probes. There is a very significant effect in one site, but no effect at all in the neighboring sites, which is a warning sign:

```
# Example of a cross-reactive probe:
locusplot("cg16517021", stats = mlma,
         windowsize = 5000, significance_line = TRUE)
```

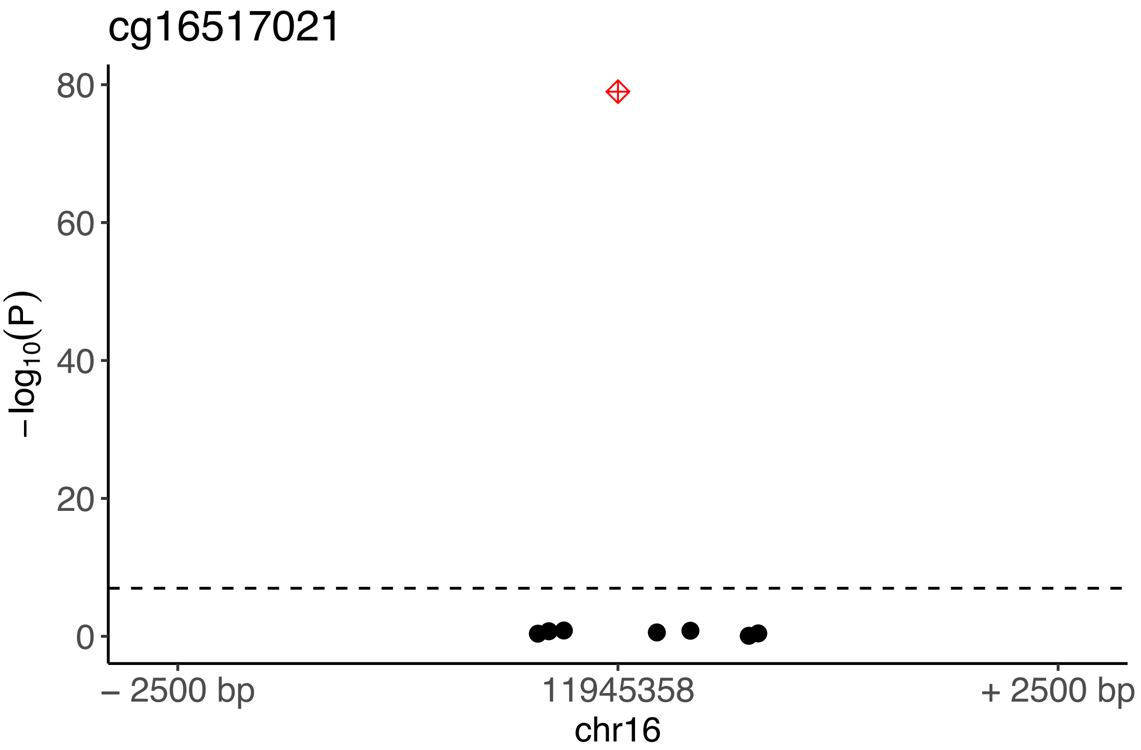

5. Associations in type I OOB channels

In the manuscript we show that several probes were significant in an EWAS on β-values derived from the out-of-band (OOB) channels. All these probes were probes with a partial match to the C9 repeat and were predicted to result in OOB sigal upon cross-hybridization. We thus found that inspecting OOB β-values can aid in identifying technical issues.

Notes

1. Associations in the OOB channels are in itself not problematic, since signals from these channels are not used in analyses. However, associations in OOB channels can help in identifying problematic sequences. In the *C9orf72* case, all OOB associations shared the same sequence, and thus helped pointing towards problematic probe sequences that also affected in-band associations.

Example We included a function to extract OOB betas/intensities from a RGset (*minfi*). Within this function, data is first normalized using the *naten* function from the *wateRmelon* package. An EWAS can then be performed on these betas using your preferred EWAS method (we prefer the OSCA method (Zhang et al. 2019)).

```
# Using example data included in minfiData:
baseDir <- system.file("extdata", package="minfiData")
samplesheet <- read.metharray.sheet(baseDir)
```

```
## [read.metharray.sheet] Found the following CSV files:
## [1] "/Users/phop2/Library/R/3.5/library/minfiData/extdata/SampleSheet.csv"
```

```
rgset <- read.metharray(samplesheet$Basename, extended=TRUE)

oob <- get_OOB(rgset, normalized = TRUE, keep = "both")
oob_betas <- oob$beta
oob_total_intensities <- oob$total
```

6. Associations between total intensity and the phenotype

An association between total signal intensity and the phenotype may indicate differences in the number of hybridization events. In that case, this indicates that there is either a copy number difference at the target locus, or that the probe cross-hybridizes to a region where there is a copy number difference.

Notes

1. Signal intensity effects may be confounded by signal saturation effects, that is, for some probes β-values near 1 or 0 (i.e. fully methylated, or fully unmethylated) tend to have lower total signal intensities than β-values near 0.5 (intermediate methylation) (Zhou et al. 2018). Thus, total signal intensity differences are especially informative when the direction of effect is opposite to what is to be expected based on signal saturation effects. For example, when β-values in the phenotype of interest are closer to 0.5, but total intensities are lower, this cannot be explained by signal saturation effects.

Examples EWAS methods normally used on β-values (or M-values) can also be used to test for associations between total signal intensities and the phenotype of interest. Extracting total signal intensities:

```
# Extract total signal intensities after normalization using dasen
rgset_dasen <- dasen(rgset)
total <- getCN(rgset_dasen)

# Extract total OOB signal intensities after normalization using dasen
# Using the function from DNAmCrosshyb
oob <- get_OOB(rgset, normalized = TRUE, keep = "both")
oob_total <- oob$total
```

7. Other issues

Finally, we note that in addition to cross-hybridization, other issues such as SNPs underlying the probe sequence could result in spurious associations. These issues are outside the scope of the current work, but we note that previously studies have considered these issues in detail (Zhou, Laird, and Shen 2017; Andrews et al. 2016). Example using population-specific SNP masking from Zhou *et al.*

```
## Select SNP-containing probes based on European population:
zhou_450k_pop_EUR <- zhou_450k_pop %>%
  dplyr::filter(MASK_snp5_EUR)
```

Furthermore, one might want to filter probes that have a low mapping quality:

```
zhou_450k %>% dplyr::filter(MASK_mapping) %>% dplyr::select(probeID, MASK_mapping) %>% head()
```

```
## # A tibble: 6 x 2
##   probeID    MASK_mapping
##   <chr>      <lgl>
## 1 cg13869341 TRUE
## 2 cg14008030 TRUE
## 3 cg12045430 TRUE
## 4 cg20826792 TRUE
## 5 cg00381604 TRUE
## 6 cg20253340 TRUE
```

References

Andrews, Shan V., Christine Ladd-Acosta, Andrew P. Feinberg, Kasper D. Hansen, and M. Daniele Fallin. 2016. "'Gap Hunting' to Characterize Clustered Probe Signals in Illumina Methylation Array Data." *Epigenetics & Chromatin* 9 (56). https://doi.org/10.1186/s13072-016-0107-z.

Mill, Jonathan, and Bastiaan T. Heijmans. 2013. "From Promises to Practical Strategies in Epigenetic Epidemiology." *Nature Reviews Genetics* 14 (8): 585–94. http://www.nature.com/nrg/journal/v14/n8/abs/nrg3405.html.

Zhang, Futao, Wenhan Chen, Zhihong Zhu, Qian Zhang, Marta F. Nabais, Ting Qi, Ian J. Deary, et al. 2019. "OSCA: A Tool for Omic-Data-Based Complex Trait Analysis." *Genome Biology* 20 (107). https://doi.org/10.1186/s13059-019-1718-z.

Zhou, Wanding, Peter W. Laird, and Hui Shen. 2017. "Comprehensive Characterization, Annotation and Innovative Use of Infinium DNA Methylation BeadChip Probes." *Nucleic Acids Research* 45 (4): e22. https://doi.org/10.1093/nar/gkw967.

Zhou, Wanding, Timothy J Triche, Peter W Laird, and Hui Shen. 2018. "SeSAMe: Reducing Artifactual Detection of DNA Methylation by Infinium BeadChips in Genomic Deletions." *Nucleic Acids Research* 46 (20): e123. https://doi.org/10.1093/nar/gky691.
